# Association of sugar intake from different sources with cardiovascular disease incidence in the prospective cohort of UK Biobank participants

**DOI:** 10.1186/s12937-024-00926-4

**Published:** 2024-02-22

**Authors:** Sylva Mareike Schaefer, Anna Kaiser, Gerrit Eichner, Mathias Fasshauer

**Affiliations:** 1 Institute of Nutritional Science, Justus-Liebig University of Giessen, Giessen, 35390 Germany; 2 Mathematical Institute, Justus-Liebig University of Giessen, Giessen, Germany; 3 Center for Sustainable Food Systems, Justus-Liebig University of Giessen, Giessen, Germany

**Keywords:** Carbohydrates, Cardiovascular disease, Ischemic heart disease, Stroke, Sugar, UK Biobank

## Abstract

**Background:**

The relation between incident cardiovascular disease (CVD) and sugar might not only depend on the quantity consumed but also on its source. This study aims to assess the association between various sources of dietary sugars and CVD incidence in the prospective population-based UK Biobank cohort.

**Methods:**

A total of 176,352 participants from the UK Biobank with at least one web-based dietary questionnaire (Oxford WebQ) for assessment of sugar intake were included in this study. Mean follow-up lasted 10.9 years (standard deviation 2.0), with 12,355 incident cases of CVD. To determine the association of free sugar (FS) and intrinsic sugar intake with incident CVD, hazard ratios (HR) were calculated using Cox proportional hazard regression models. FS intake from beverages and beverage subtypes, i.e., soda/fruit drinks, juice, milk-based drinks, and tea/coffee, as well as from solid foods and solids subtypes, i.e., treats, cereals, toppings, and sauces, was included as penalised cubic splines.

**Results:**

FS intake showed a J-shaped relationship with CVD risk, reaching the lowest HR (HR-nadir) at 9 %E, while intrinsic sugars displayed a non-linear descending association, with the HR-nadir at 14 %E. FS in beverages demonstrated a significant linear relationship with CVD with the HR-nadir at 3 %E, while FS in solids exhibited a significant non-linear U-shaped relationship with the HR-nadir at 7 %E. Within the beverage subtypes, soda/fruit drinks displayed a linear relationship, as did to a lesser extent FS in milk-based drinks and tea/coffee. Juice, however, showed a significant U-shaped relationship with CVD risk. Among solid foods subtypes, FS in treats had a J-shaped relation with the HR-nadir at 5 %E, and FS in cereals showed a linear association. In comparison, FS in toppings and sauces exhibited a U-shaped pattern with HR-nadir at 3 %E and 0.5 %E, respectively. All major results remained similar in various sensitivity analyses and were more robust for ischemic heart disease compared to stroke.

**Conclusions:**

Only some sources of FS exhibit a robust positive association with CVD incidence. Public health efforts aiming at the reduction of CVD risk should prioritise the reduction of sugary beverages with an emphasis on soda/fruit drinks.

**Supplementary Information:**

The online version contains supplementary material available at 10.1186/s12937-024-00926-4.

## Introduction

Cardiovascular disease (CVD) remains a major contributor to global mortality, accounting for approximately 32 % of all deaths worldwide [1]. CVD encompasses a spectrum of pathological conditions that affect the cardiovascular system, including diseases such as ischemic heart disease (IHD) and stroke [2, 3]. Advanced age, male sex, and genetic predisposition are important non-modifiable risk factors [3]. Major modifiable risk factors are an unhealthy diet, inadequate physical activity, harmful alcohol consumption, and tobacco use [3]. These risk factors can cause hypertension, hyperlipidaemia, type 2 diabetes mellitus, and obesity [1].

There is growing evidence that a high sugar diet is an important risk factor for CVD [2, 4] and several mechanisms have been proposed. Thus, calorie intake from sugary beverages induces less satiety, incomplete compensatory reduction in energy intake at subsequent meals, as well as a positive energy balance in humans [5]. Furthermore, dietary sugars increase hepatic de novo lipogenesis with concomitant non-alcoholic fatty liver disease (NAFLD) [6] and NAFLD is associated with increased long-term risk of CVD [7]. Further potential mechanisms linking high sugar consumption and CVD risk include increased sympathetic activity via the ventromedial hypothalamus [8] and reactive oxygen species-mediated oxidative stress [9].

Sugars include both mono- and disaccharides [10] and are classified into two different types according to the World Health Organization (WHO): free sugars (FS) and intrinsic sugars [11]. FS are all monosaccharides and disaccharides added to foods by the manufacturer, cook, or consumer, plus sugars naturally present in honey, syrups, and fruit juices [11]. In contrast, intrinsic sugars include sugars from fruit and vegetables, as well as lactose and galactose in dairy products [11]. According to the WHO, FS consumption should be reduced throughout the life course [11]. The WHO provides a strong recommendation that FS intake should be reduced to less than 10 % of total energy (%E) intake [11]. It conditionally recommends a further reduction of FS consumption to below 5 %E [11]. This corresponds to about 50 g and 25 g FS per day, respectively, in the case of a 2,000-kcal diet [11]. In agreement with the WHO, the National Health Service England recommends a daily FS intake of less than 30 g for adults [12].

High consumption of sugar, particularly in the form of sugar-sweetened beverages (SSB), is strongly associated with an increased risk of CVD [13–15]. In contrast, fruits and vegetables appear to have beneficial effects on cardiovascular health [16, 17]. However, no large study with more than 10,000 participants has systematically assessed the association between FS from all relevant sources which are summarised in Fig. S1 with CVD risk so far. Furthermore, no study has evaluated the relationship between intrinsic sugars and incident CVD. Therefore, the associations of the intake of FS, intrinsic sugars, and various FS subtypes with incident CVD were analysed in a large, well-characterised population of 176,352 UK Biobank participants using penalised cubic splines to allow non-linear predictor effects. We hypothesised that the relationship between FS and CVD risk depends on the source of FS with a positive association found for beverages, but not for solid foods, similar to recent findings from our group studying all-cause mortality [18], incident depression [19], and incident dementia [20].

## Methods

### Study design, participants, and exclusion criteria

The UK Biobank study is a prospective cohort study in which more than half a million participants aged 37 to 73 were recruited across the UK between 2006 and 2010 [21].

Written informed consent was obtained from all participants at baseline and ethical approval for the UK Biobank study was granted by the North West Multicentre Research Ethics Committee [21]. Participants from the UK Biobank pilot phase were removed from the analysis (*n* = 3,794; Fig. S2) since questions for covariates of the present study were different in some instances in the pilot as compared to the later cohort, e.g., professional qualifications were not assessed within the pilot. Since the intake of sugar and sugar subtypes was assessed based on the previous 24-h dietary intakes (Oxford WebQ) [22], participants without completing at least one dietary questionnaire had to be excluded (*n* = 287,620; Fig. S2). Seven exclusion criteria were applied similar to three recent studies from our group [18–20] (removed participants *n* = 34,593) and are depicted in Fig. S2.

### Exposure assessment

Participants were asked to complete the Oxford WebQ, a web-based dietary recall tool that assesses food and beverage intake from the previous day. The Oxford WebQ provides quantitative data on 206 food and 32 beverage items, covering major foods consumed in the UK [22, 23]. While it addresses general food items, specific brands are not considered, except for occasional examples, e.g., “Chocolate bars (e.g. Crunchie, Snickers)”. The Oxford WebQ is susceptible to person-specific biases, influenced by factors like age, sex, and body mass index [24–26]. Further person-specific biases include reactivity, memory, and social desirability bias [25, 27]. Moreover, inherent characteristics of the assessment tool, e.g., inadequate strategies for data collection or visual support, can also contribute to nutritional assessment errors [27]. Despite potential biases, the Oxford WebQ has been validated against accelerometry-estimated energy expenditure and biomarkers, showing good performance compared to traditional 24-hour interviewer-led dietary recalls [27]. Since underreporting of fat and carbohydrates with food records and 24-h recalls has been repeatedly demonstrated [28, 29], participants with significant underreporting of energy intake, i.e., total energy intake below 1.1 times basal metabolic rate assessed by the Oxford equation [30] minus 500 kcal, were excluded. Performance of the Oxford WebQ improves when multiple questionnaires are filled out [27], but this comes at the cost of losing more than a third of the sample size, i.e., 67,036 participants. To maintain a larger sample, main analyses were conducted on the total cohort with at least one Oxford WebQ, and additional sensitivity analyses were performed on participants with two or more questionnaires.

The intake of sugar and sugar subtypes from beverages and solids was calculated using the Oxford WebQ data similar to described in three previous reports from our group [18–20]. In brief, the definition of sugary beverages included soda/fruit drinks, pure juice, milk-based drinks, and tea/coffee with added sugar, whereas treats, breakfast cereals, toppings, and sauces were defined as subtypes of sugary solids. The size of a standard portion of these food items was taken from the UK Food Standards Agency [31] and respective product labels. To obtain the total consumed amount of sugar in each beverage and solids subtype, the reported consumption frequency of each food item was multiplied by the estimated content of this sugar subtype in that food item per serving. To calculate sugar subtype intake in %E, the intake in g/d was multiplied by 17 kJ/g * 100 % / total energy in kJ/d according to Willett and colleagues [32]. The amount of intrinsic sugars consumed was calculated from the difference between total sugars and FS. For all participants who completed more than one Oxford WebQ, their mean intake measured in %E of all sugar subtypes was used in all primary and sensitivity analyses except for Fig. S7 where only the first completed Oxford WebQ was considered.

### Outcome assessment

The primary outcome of the study was incident CVD defined as International Classification of Disease 10^th^ revision (ICD-10) codes I21-25, I60, I61, I63, and I64. Subgroup analyses were performed for IHD (ICD-10 codes I21-I25) and stroke (ICD-10 codes I60, I61, I63, and I64). Outcomes were defined as the first occurrence of these ICD-10 codes across self-report at baseline assessment, primary care, hospital in-patient records, and death record data in the UK Biobank [33]. In order to calculate the follow-up time, the date of the first dietary assessment was subtracted from the date of the first diagnosis of any CVD event, loss-to-follow-up, death, or censoring, whichever came first. In case of more than one diagnosis, the shortest duration to any diagnosis was used.

### Statistical analyses

Data analysis was performed with R version 4.3.2 (R Core Team, Vienna, Austria) [34] as described recently [19]. In brief, the hazard ratios (HR) for incident CVD were assessed with Cox proportional hazard regression multivariate nutrient density models [32] including %E intake of sugar from different sources and total energy intake as penalised cubic splines with their degrees of freedom set to 4. Splines are used to produce curve shapes with a high degree of flexibility when fitting the model [35]. Penalised cubic splines impose a penalisation upon the piecewise polynomial components to optimise the model fit [35]. They are useful to identify complex patterns without the user having to specify various parameters [35]. A directed acyclic graph (produced with the R package DAGitty [36] that shows hypothesized causal relationships underlying the association between sugar and CVD incidence was used to identify an appropriate set of confounding variables to assess an unconfounded effect estimate. Hence, the following variables were selected as covariates in the analysis: age, energy intake, highest qualification, physical activity (MET per week), sex, and smoking (Fig. S3). If a significant violation of the assumption of hazard proportionality was detected using scaled Schoenfeld residuals, the respective covariates were stratified in the final models. In each analysis, the estimation of the lowest value of the hazard ratio (HR) on the sugar intake axis, called the HR–nadir, was restricted to the range of sugar intake from zero to the 99th percentile of the observed intakes. To simplify the presentation, the HR was then rescaled to a value of 1 at its nadir. In all Cox proportional hazard regression models, the HRs are presented along with their corresponding pointwise 95 % confidence intervals. The analysis of each penalised cubic spline was divided into the linear effect (p^lin^) and the non-linear effect (p^non-lin^), as recently described [37]. In all analyses, a *p-value* of <0.05 was considered statistically significant. No further interpretation of the HR-nadir or of other individual HRs was performed if both p^lin^ and p^non-lin^ were non-significant.

### Sensitivity analyses

To evaluate the robustness of the results, we performed several sensitivity analyses similarly as described in recent studies [18, 19, 38]: To address reverse causation, participants who were diagnosed with CVD or lost to follow-up within two years of joining the study (landmark analysis) were excluded. Participants with unintentional weight loss were also removed in another sensitivity analysis as this might be a sign, e.g., of malignant diseases, chronic organ failure, frailty, and psychological disorders [39]. To ensure more representative consumption data, participants who reported at least once having had an atypical diet on the previous day were also excluded. To focus on nutrient intake closest to the baseline assessment, the analyses were repeated using only the first Oxford WebQ questionnaire. Additionally, a diet quality score was calculated by combining five dietary components: fat, fruit, vegetables, red meat, and processed meat consumption in order to control for potential residual confounding due to dietary factors as described by Anderson and colleagues [38]. Furthermore, participants who filled out only one Oxford WebQ were removed from the analysis to address potential variation, i.e., lower reproducibility in sugar intake based on a single Oxford WebQ [27]. Lastly, CVD outcomes were divided into the subgroups ischemic heart disease and stroke to assess whether associations remained consistent.

## Results

An overview of all main results on associations of sugar subtypes with CVD risk is shown in Table S1.

### Baseline data of UK Biobank participants

In total, 176,352 participants were included in the present study (Fig. S2). The baseline characteristics of the studied population in total and in subgroups of FS intake defined by %E quintiles are presented in Table 1. Mean (standard deviation (SD)) age of the study cohort at completion of the first Oxford WebQ was 57 [8] years and 58.5 % of the participants were female. The follow-up period was 10.9 (2.0) years, i.e., 1.9 million person-years. Out of the total of 12,355 cases of CVD, there were 9,950 cases of IHD and 3,066 cases of stroke, i.e., 661 participants having both diagnoses.

**Table 1:** Baseline characteristics of the UK Biobank cohort^a^

Parameters	Total cohort (*n*=176,352)	FS intake (%E) split by quintiles				
		0.0 to 6.8 (*n*=35,271)	6.8 to 9.5 (*n*=35,270)	9.5 to 12.1 (*n*=35,270)	12.1 to 15.4 (*n*=35,270)	15.4 to 77.5 (*n*=35,271)
**Characteristics**						
Age at completion of first Oxford WebQ (years)	57 (8)	57 (8)	58 (8)	58 (8)	58 (8)	57 (8)
BMI (kg/m^2^)	26.5 (4.3)	26.7 (4.4)	26.5 (4.3)	26.4 (4.2)	26.3 (4.3)	26.6 (4.4)
Energy (kJ/d)	9,079 (2,307)	8,515 (2,246)	8,994 (2,236)	9,214 (2,253)	9,337 (2,287)	9,334 (2,407)
Ethnic background						
- White	169,902 (96.3)	34,095 (96.7)	34,242 (97.1)	34,158 (96.8)	34,094 (96.7)	33,313 (94.4)
- Mixed, Asian, Black, Chinese, and other	6,450 (3.7)	1,176 (3.3)	1,028 (2.9)	1,112 (3.2)	1,176 (3.3)	1,958 (5.6)
General health status						
- Poor	3,636 (2.1)	637 (1.8)	545 (1.5)	595 (1.7)	714 (2.0)	1,145 (3.2)
- Fair	26,538 (15.1)	5,299 (15.0)	4,918 (13.9)	4,875 (13.8)	5,179 (14.7)	6,267 (17.8)
- Good	107,977 (61.2)	21,579 (61.2)	21,624 (61.3)	21,943 (62.2)	21,780 (61.8)	21,051 (59.7)
- Excellent	38,201 (21.7)	7,756 (22.0)	8,183 (23.2)	7,857 (22.3)	7,597 (21.5)	6,808 (19.3)
Highest qualification						
- None of the below	13,355 (7.6)	2,914 (8.3)	2,485 (7.0)	2,523 (7.2)	2,515 (7.1)	2,918 (8.3)
- National exams at age 16 years	26,565 (15.1)	5,385 (15.3)	5,079 (14.4)	5,145 (14.6)	5,235 (14.8)	5,721 (16.2)
- Vocational qualifications or optional national exams at ages 17-18 years	31,197 (17.7)	6,390 (18.1)	6,086 (17.3)	6,009 (17.0)	6,007 (17.0)	6,705 (19.0)
- Professional	27,196 (15.4)	5,266 (14.9)	5,237 (14.8)	5,587 (15.8)	5,518 (15.6)	5,588 (15.8)
- College or University	78,039 (44.3)	15,316 (43.4)	16,383 (46.5)	16,006 (45.4)	15,995 (45.4)	14,339 (40.7)
History of cancer	14,628 (8.3)	2,829 (8.0)	2,872 (8.1)	2,970 (8.4)	3,030 (8.6)	2,927 (8.3)
History of mental illnesses	11,590 (6.6)	2,292 (6.5)	2,161 (6.1)	2,099 (6.0)	2,276 (6.5)	2,762 (7.8)
Hypertension	36,942 (21.0)	7,949 (22.5)	7,334 (20.8)	7,248 (20.6)	7,147 (20.3)	7,264 (20.6)
Physical activity (MET-min/week)	4,133 (2,647)	4,068 (2,654)	4,112 (2,566)	4,139 (2,560)	4,145 (2,597)	4,202 (2,848)
Sex – female	103,136 (58.5)	20,720 (58.7)	20,974 (59.5)	20,731 (58.8)	20,669 (58.6)	20,042 (56.8)
Smoking status						
- Never	102,774 (58.3)	18,383 (52.1)	19,970 (56.6)	20,912 (59.3)	21,752 (61.7)	21,757 (61.7)
- Previous	60,959 (34.6)	14,043 (39.8)	12,983 (36.8)	12,178 (34.5)	11,222 (31.8)	10,533 (29.9)
- Occasional	4,267 (2.4)	1,018 (2.9)	874 (2.5)	816 (2.3)	778 (2.2)	781 (2.2)
- Current <10 cigarettes per day	2,221 (1.3)	477 (1.4)	400 (1.1)	374 (1.1)	450 (1.3)	520 (1.5)
- Current 10 to 14 cigarettes per day	1,894 (1.1)	384 (1.1)	327 (0.9)	316 (0.9)	341 (1.0)	526 (1.5)
- Current 15 to 19 cigarettes per day	1,661 (0.9)	353 (1.0)	278 (0.8)	273 (0.8)	297 (0.8)	460 (1.3)
- Current ≥20 cigarettes per day	2,576 (1.5)	613 (1.7)	438 (1.2)	401 (1.1)	430 (1.2)	694 (2.0)
Total household income per year (k£)						
- <18	22,694 (12.9)	4,302 (12.2)	4,142 (11.7)	4,292 (12.2)	4,627 (13.1)	5,331 (15.1)
- 18 to <31	38,072 (21.6)	7,242 (20.5)	7,431 (21.1)	7,617 (21.6)	7,772 (22.0)	8,010 (22.7)
- 31 to <52	46,119 (26.2)	9,112 (25.8)	9,321 (26.4)	9,290 (26.3)	9,199 (26.1)	9,197 (26.1)
- 52 to <100	40,258 (22.8)	8,556 (24.3)	8,434 (23.9)	8,162 (23.1)	7,916 (22.4)	7,190 (20.4)
- ≥100	12,055 (6.8)	2,773 (7.9)	2,657 (7.5)	2,450 (6.9)	2,280 (6.5)	1,895 (5.4)
- Unknown	17,154 (9.7)	3,286 (9.3)	3,285 (9.3)	3,459 (9.8)	3,476 (9.9)	3,648 (10.3)
Townsend deprivation index	-1.7 (2.8)	-1.5 (2.9)	-1.7 (2.8)	-1.7 (2.8)	-1.8 (2.8)	-1.5 (2.9)
**Dietary sugar subtype intake in %E**						
Intrinsic sugars	13.0 (5.7)	14.4 (6.5)	13.7 (5.6)	13.1 (5.3)	12.5 (5.1)	11.4 (5.3)
FS	11.4 (5.6)	4.5 (1.7)	8.2 (0.8)	10.8 (0.7)	13.6 (1.0)	19.6 (4.3)
FS beverages	4.7 (4.6)	1.1 (1.4)	2.6 (2.1)	3.9 (2.5)	5.6 (3.0)	10.4 (5.8)
- Soda/fruit drinks	1.6 (3.2)	0.2 (0.6)	0.5 (1.2)	0.9 (1.7)	1.6 (2.4)	4.6 (5.4)
- Juice	2.1 (2.8)	0.6 (1.2)	1.4 (1.8)	2.1 (2.2)	2.7 (2.6)	3.8 (4.0)
- Milk-based drinks	0.3 (0.9)	0.1 (0.5)	0.2 (0.7)	0.3 (0.9)	0.4 (1.0)	0.6 (1.3)
- Tea/coffee	0.6 (1.6)	0.2 (0.6)	0.3 (0.9)	0.5 (1.2)	0.7 (1.6)	1.4 (2.6)
FS solids	6.6 (3.5)	3.4 (1.8)	5.6 (2.1)	6.9 (2.5)	8.1 (3.0)	9.2 (4.1)
- Treats	4.4 (3.0)	2.1 (1.6)	3.6 (2.0)	4.5 (2.4)	5.3 (2.8)	6.3 (3.8)
- Cereals	0.5 (0.8)	0.3 (0.6)	0.5 (0.7)	0.5 (0.8)	0.6 (0.8)	0.6 (0.9)
- Toppings	1.2 (1.6)	0.4 (0.9)	0.9 (1.4)	1.3 (1.6)	1.6 (1.8)	1.7 (2.0)
- Sauces	0.3 (0.4)	0.2 (0.4)	0.3 (0.4)	0.3 (0.4)	0.3 (0.4)	0.3 (0.4)
**Number of Oxford WebQs**	2.2 (1.2)	2.0 (1.1)	2.3 (1.2)	2.4 (1.2)	2.3 (1.2)	2.0 (1.1)

^a^Categorical variables are summarised as frequencies (percentages) and continuous variables as mean (SD)

*CVD* Cardiovascular disease, *FS* Free sugars, *kJ* Kilojoules, *MET* Metabolic equivalent of task, *%E* Percentage total energy, *SD* Standard deviation

### Main analyses

#### FS and intrinsic sugars

**Fig. 1:**
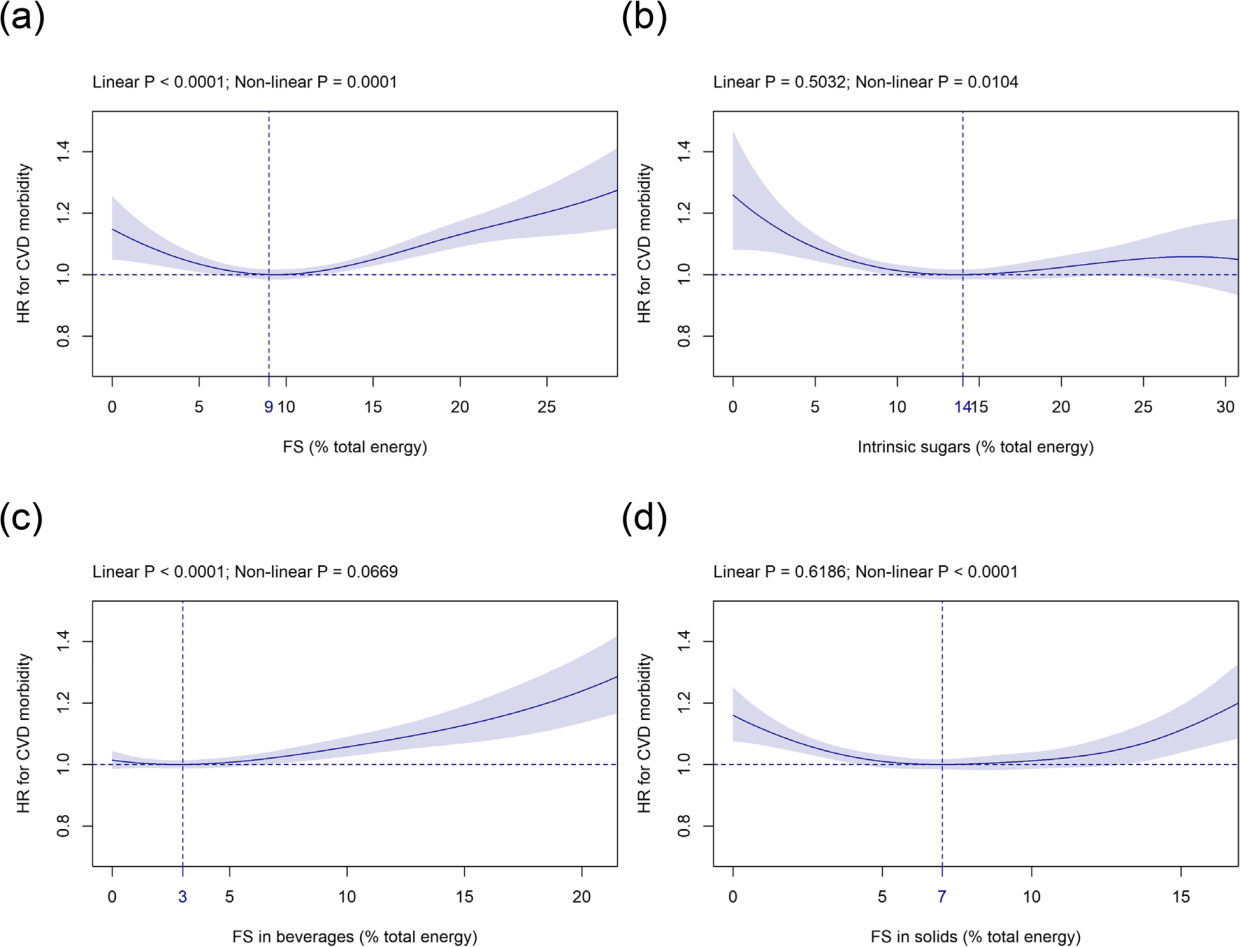
Association of (**a**) FS, (**b**) intrinsic sugars, (**c**) FS in beverages, and (**d**) FS in solids intake (all %E) with CVD risk. Models are adjusted for age (split by quintiles), energy intake (penalised cubic splines), highest qualification (none of the below, national exams at age 16 years, vocational qualifications or optional national exams at ages 17-18 years, professional, College or University), physical activity (metabolic equivalent of task-minutes per week derived from the Oxford WebQ; split by quintiles), sex (female, male), and smoking status (never, previous, current occasional, current <10, 10 to 14, 15 to 19, ≥20 cigarettes per day). Covariates not fulfilling the proportional hazard assumption are stratified. The vertical line indicates the HR-nadir. Abbreviations: CVD, Cardiovascular disease; FS, Free sugars; HR, Hazard ratio; %E, Percentage total energy

As shown in Table 1, the mean (SD) consumption of FS and intrinsic sugars was 11.4 (5.6) %E and 13.0 (5.7) %E, respectively. FS intake was significantly linked to HR for CVD in a J-shaped fashion with the HR-nadir observed at an intake level of 9 %E (Fig. 1a). In comparison to intake at the HR-nadir, the HR increased to 1.13 (1.09 to 1.17) at 20 %E (Fig. 1a). Intrinsic sugars were related with CVD risk in a non-linear descending manner with the HR-nadir at 14 %E and an increased HR of 1.26 (1.08 to 1.47) at 0 %E (Fig. 1b).

#### FS in beverages and FS in solids

Mean (SD) intake of FS in beverages and FS in solids was 4.7 (4.6) %E and 6.6 (3.5) %E, respectively (Table 1). For FS in beverages, a significant linear relation could be detected with the HR-nadir at 3 %E and an increase to 1.06 (1.03 to 1.09) and 1.24 (1.13 to 1.35) at 10 %E and 20 %E, respectively (Fig. 1c). For FS in solids the relation was slightly U-shaped and the HR-nadir was detected at 7 %E and increased to 1.16 (1.07 to 1.25) at 0 %E (Fig. 1d).

#### FS in beverage subtypes

**Fig. 2:**
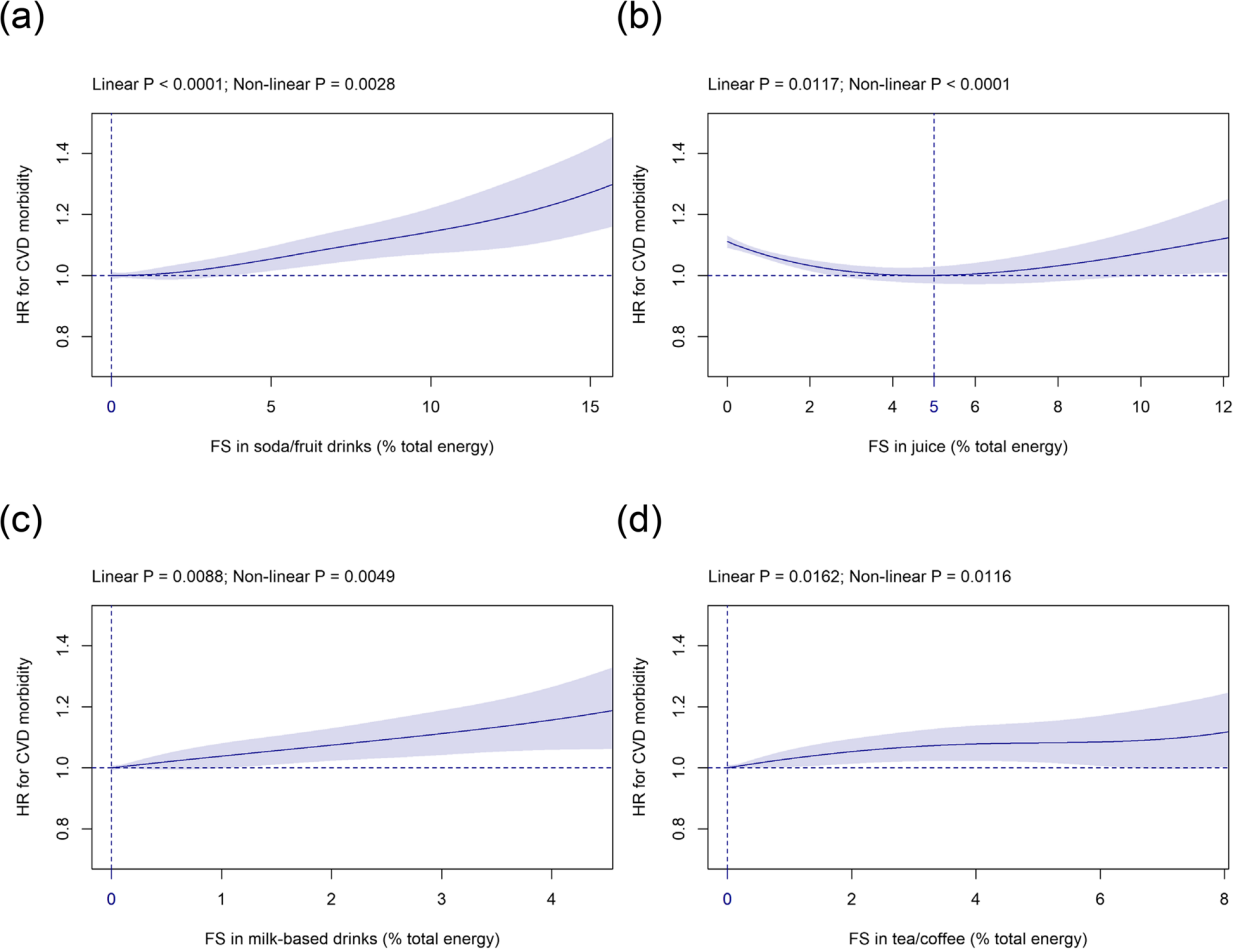
Association of FS in (**a**) soda/fruit drinks, (**b**) juice, (**c**) milk-based drinks, and (**d**) tea/coffee (all %E) with CVD risk. Models are adjusted and presented as indicated in Fig. 1. Abbreviations: %E, Percentage total energy

Mean (SD) intake of FS in beverage subtypes was as follows: soda/fruit drinks 1.6 (3.2) %E, juice 2.1 (2.8) %E, milk-based drinks 0.3 (0.9) %E, and tea/coffee 0.6 (1.6) %E (Table 1). FS in soda/fruit drinks showed a significant linear association with CVD risk with the HR nadir found at 0 %E and a HR of 1.14 (1.07 to 1.22) and 1.27 (1.14 to 1.42) at 10 %E and 15 %E, respectively (Fig. 2a). FS in juice were significantly related with HR for CVD in a U-shaped fashion with HR-nadir observed at 5 %E and HRs of 1.11 (1.09 to 1.13) and 1.07 (1.00 to 1.15) at 0 %E and 10 %E FS, respectively (Fig. 2b). FS in milk-based drinks (Fig. 2c) and FS in tea/coffee (Fig. 2d) were both significantly linearly associated with CVD risk and the HR-nadir was at 0 %E for both.

#### FS in solids subtypes

**Fig. 3:**
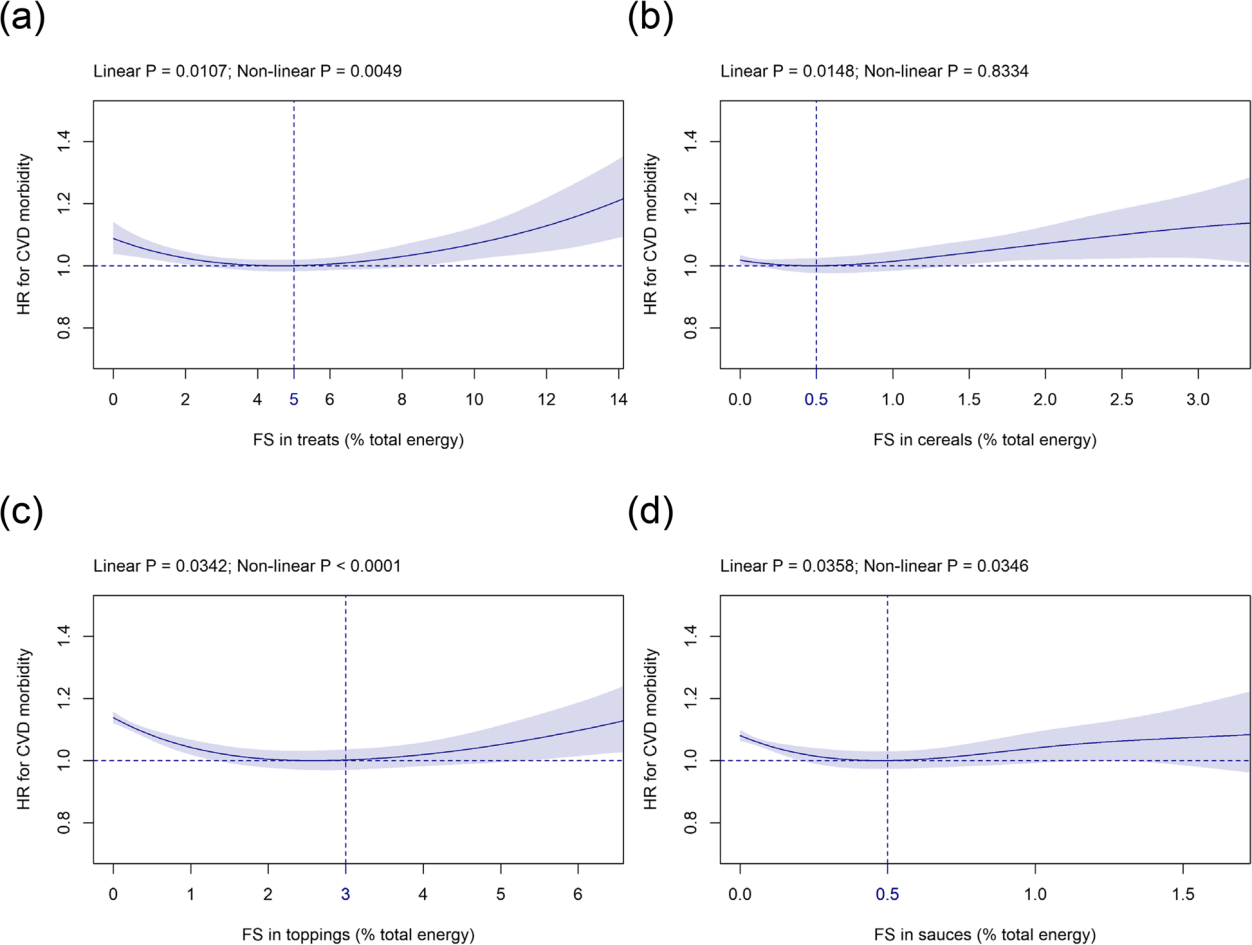
Association of FS in (**a**) treats, (**b**) cereals, (**c**) toppings, and (**d**) sauces (all %E) with CVD risk. Models are adjusted and presented as indicated in Fig. 1. Abbreviations: %E, Percentage total energy

Mean (SD) intake of FS in solids subtypes was as follows: treats 4.4 (3.0) %E, cereals 0.5 (0.8) %E, toppings 1.2 (1.6) %E, and sauces 0.3 (0.4) %E (Table 1). The relation between FS in treats and CVD risk was J-shaped with the HR-nadir at 5 %E (Fig. 3a) while the relation was U-shaped for FS in toppings and sauces with the HR-nadir at 3 %E and 0.5 %E (Fig. 3c, d), respectively. FS in cereals showed a significant linear association with incident CVD with the HR-nadir at 0.5 %E (Fig. 3b).

### Sensitivity analyses

#### FS and intrinsic sugars

The association between FS and HR for CVD remained significant in all sensitivity analyses (Fig. S4a to S9a). Analysing the subgroups of CVD, the shape of the relation between FS and CVD risk was similar for IHD (Fig. S10a) but changed for stroke: the HR-nadir was at 11 %E and a considerable increase of the HR at 0 %E to 1.31 (1.10 to 1.56) was found (Fig. S10b). The relation between intrinsic sugars and CVD risk remained significant in all sensitivity analyses except for the removal of participants with a non-typical diet (Fig. S6b) and when the subgroup of stroke was analysed (Fig. 10d).

#### FS in beverages and FS in solids

The association between FS in beverages and CVD was virtually identical in all sensitivity analyses (Fig. S4c to S9c) and subgroup analyses (Fig. S10e, f). For FS in solids the significant U-shaped relation remained significant in the subgroup analyses and in all but one sensitivity analysis (Fig. S9d).

#### FS in beverage subtypes

The findings for FS in soda/fruit drinks, juice, milk-based drinks and tea/coffee were robust in all sensitivity analyses except for FS in milk-based drinks who were not significantly related to stroke incidence (S10n).

#### FS in solids subtypes

Findings on FS in solids subtypes changed in the following sensitivity analyses: FS in treats were linearly related to CVD when only the first Oxford WebQ was used (Fig. S7i), lost significance when only participants with more than 1 Oxford WebQ were included in the analysis (Fig. S9i), and for stroke the relation became U-shaped (Fig. S10r). FS in cereals remained significantly associated with CVD risk only in two sensitivity analyses (Fig. S5j, S8j). FS in toppings remained significantly associated with CVD morbidity in all sensitivity analyses (Fig. S4k to S9k) and in the subgroup analysis (Fig. S10u, v). FS in sauces remained significantly associated with CVD in two sensitivity analyses (Fig. S6l, S8l). In subgroup analysis, FS in sauces were significantly associated with IHD risk (Fig. 10w) but not with incident stroke (Fig. 10x).

## Discussion

### Principal findings

This study aims to assess the relationship between FS and incident CVD in a large prospective cohort. The analysis comprehensively examines FS intake from all relevant sources, including beverages and solids, using penalised cubic splines to account for non-linear relationships. FS intake show a J-shaped relationship with CVD risk, reaching the HR-nadir at 9 %E, while intrinsic sugars display a non-linear descending association, with the HR-nadir at 14 %E. There is a significant linear relationship between FS in beverages and HR for incident CVD, while a U-shaped relation can be detected for FS in solids with the lowest risk at 7 %E. FS in soda/fruit drinks, milk-based drinks, and tea/coffee are significantly linearly related with the HR-nadir at 0 %E, while juice shows a U-shaped association with the lowest HR at 5 %E. FS in treats show a J-shaped relation with the HR-nadir at 5 %E while FS in cereals are linearly associated with CVD risk. FS in toppings and sauces exhibit a U-shaped pattern with the HR-nadir at 3 %E and 0.5 %E, respectively. Major findings remain robust in various sensitivity analyses. Since results including the diet quality score as a covariate are not substantially different as compared to the main analyses, the association between FS subtypes and CVD risk cannot be simply explained by overall diet quality.

### Comparison with other studies

Other studies focusing on added sugars or FS have reached inconclusive results [40–43]. Our findings align with a Swedish report that observes a U-shaped trend for added sugars and incident stroke with the lowest risk for consumers in the 7.5 %E to 10 %E group and an increasing risk among the lowest and highest intake groups [40]. In a meta-analysis, a threshold for harm is identified for added sugars at 13 %E in relation to CVD mortality; however, the evidence is rated as low to very low certainty [41]. A study in 109,034 women from the Women’s Health Initiative shows that the consumption of added sugars of ≥15.0 %E is positively associated with total CVD and IHD risk [42]. However, another recent study in Canadians does not show conclusive associations for FS above vs. below a threshold of 10 %E [44]. In a previous well-conducted study in UK Biobank participants, each 5 %E increment of FS intake is positively associated with CVD, IHD, and stroke incidence [43]. Taking these and the current results into consideration, FS intake appears not to be linearly related to CVD risk but limiting FS consumption to no more than 10 %E might be beneficial for CVD prevention.

In the present study, FS in beverages are significantly associated with CVD, IHD, and stroke incidence in a linear way. To the best of our knowledge, only one small study (*n* = 8,422) has assessed the association of FS in liquid foods with CVD incidence [44]. CVD risk for FS intake above as compared to below a threshold of 5 %E in liquid foods is numerically higher but does not reach statistical significance [44]. Differences in results may be well attributable to the much larger sample size (*n* = 176,352) and the use of splines in the present analysis.

Within beverage subtypes, FS in soda/fruit drinks are significantly associated with CVD, IHD, and stroke incidence in a linear way in the present analysis. Consistent with our findings, a network meta-analysis of 21 cohort studies shows that the consumption of SSB is associated with a 14 % higher risk of CVD and a 13 % higher risk of stroke, respectively [42]. Results from the Women's Health Initiative also show an elevated risk of CVD and stroke with an intake of ≥1 serving of SSB per day [42]. In our study, the intake of FS in juice is significantly related to CVD incidence in a U-shaped fashion with the HR-nadir at 5 %E and with an 11 % higher risk in non-consumers. In CVD subgroups, the association remains significant for both IHD and stroke. Scheffers and co-workers observe a significant association of fruit juice intake up to 7 glasses per week with a reduced risk of CVD, but not beyond ≥8 glasses [45]. A significantly decreased risk of stroke is observed at intake levels of 1 to 4 and 4 to 8 glasses juice per week as compared to non-drinkers [45]. Our study is the first to elucidate the association between FS in milk-based drinks and FS added to tea/coffee on the one hand and CVD risk on the other hand. FS in milk-based drinks and FS added to tea/coffee show a linear association with CVD risk similar to FS in soda/fruit drinks. Assessing the subgroups of CVD, FS in milk-based drinks show a similar association for IHD and no significant relation with stroke. In contrast, FS in tea/coffee are significantly related with both IHD and stroke. Combined, these data and published evidence suggest that the association between FS and incident CVD depends on beverage type with a significant linear relationship observed in soda/fruit drinks and to a lesser extent in milk-based drinks and tea/coffee. In contrast, consuming a low to moderate amount of juice is not associated with an elevated risk of CVD and may even have a protective effect. This potential link should be assessed in further studies.

In the current report, FS in solids show a significant non-linear U-shaped association with the HR-nadir at 7 %E. In the subgroup analysis, the association for FS in solids and HR remains significant for both IHD and stroke. Using a different approach, Dasgupta and co-workers find a 34 % higher HR for CVD in men aged 55-75 years who consume more compared to men who consume less than 5 %E FS from solids [44]. In our study, FS in treats exhibit a J-shaped relation, FS in cereals a linear relation and FS in toppings and sauces are related in a U-shaped manner with HR for CVD. In agreement with our results, Janzi and co-workers [40] show that the highest risk of stroke and coronary events is found in the intake group of treats with ≤2 servings/week, however, they detect no association for toppings intake. Taken together, these results indicate that a linear association between FS intake and CVD risk can only be observed for beverages, especially soda/fruit drinks, milk-based drinks and FS added to tea/coffee and only to a smaller extent for FS in cereals. Different associations of FS in beverages and solids with CVD could be in part explained by faster gastric emptying of sugary liquids [46, 47]. More rapid absorption of FS from beverages triggers less satiety which leads to an incomplete compensatory reduction in energy intake at subsequent meals and a positive energy balance [5].

The present study is the first to show that intrinsic sugars are significantly associated with CVD risk in a non-linear descending way. Intrinsic sugars can be found in high amounts incorporated within the structure of intact fruit and vegetables or as naturally present lactose and galactose in dairy products [11]. It is important to note in this context that the CVD risk is reduced by 28 % in participants with an intake of 800 g per day of fruit and vegetables combined as compared to no intake in a large meta-analysis of 12 studies [48]. In agreement with these findings, comparing the highest to the lowest category of intake, an inverse association of vegetable and fruit consumption with risk of IHD and stroke is found in a meta-analysis of 123 studies [49]. In contrast, milk and dairy-product intake is not significantly related to CVD risk in a meta-analysis of 17 studies [50]. Takings these findings into consideration, intrinsic sugars are not or even inversely related to CVD risk and a moderate consumption may be beneficial for CVD prevention. It remains to be elucidated whether intrinsic sugars per se are neutral in contrast to FS concerning CVD risk or whether adverse effects of this sugar subtype are neutralised or even overcompensated by other beneficial ingredients and/or the plant matrix of intrinsic sugar-rich sources [51].

Some differences are observed between IHD and stroke. Thus, we find a significant non-linear descending association of intrinsic sugars with IHD but not with stroke risk. FS in milk-based drinks and in sauces are only significantly linearly related with incident IHD but not with stroke.

These different associations might be in part explained by different pathophysiologic mechanisms of IHD and stroke. Thus, atherosclerotic plaques in coronary arteries are the most common cause of IHD with plaque rupture and concomitant thrombosis leading to myocardial infarction [52]. In contrast, strokes are caused by a broader range of mechanisms including thrombotic and embolic events and they can be ischemic or haemorrhagic [52]. Further studies need to elucidate which mechanisms contribute to the different associations observed in the current analysis.

### Strengths and limitations

The strengths of the current study encompass a prospective cohort design, a large sample size, comprehensive characterisation of participants, a long follow-up period >10 years, systematic analyses of sugar consumption by sugar subtype, and the use of penalised cubic splines to allow non-linear relations. Some limitations of our findings have to be acknowledged. These include potential residual confounding, measurement errors in assessing the exposure variables, as well as the presence of potential confounders, mediators, and further covariates not included in the models which might significantly and independently affect the current results. It should be noted that all consumption data in this study were self-reported and not independently assessed. Participants recruited to the UK Biobank are mostly of white European ancestry and are typically healthier than the overall population [53]. Therefore, our findings may not be generalisable and further research in other populations is warranted.

## Conclusions

The association with incident CVD varies depending on the source of the sugars. Thus, FS and FS in treats are related to CVD risk in a J-shaped fashion. FS in beverages, FS in soda/fruit drinks, and to a lesser extent FS in milk-based drinks, FS in tea/coffee, and FS in cereals are significantly linearly associated with incident CVD. Furthermore, FS in solids, FS in juice, FS in toppings, and FS in sauces are significantly related to CVD risk in a U-shaped fashion. In contrast, a significant non-linear descending association of intrinsic sugars with CVD risk is found.

In sensitivity analyses, these relations are more robust for ischemic heart disease as compared to stroke. To improve CVD risk reduction strategies, public health efforts should prioritise the reduction of beverages with an emphasis on soda/fruit drinks when targeting different FS subtypes. Additional prospective studies are needed to investigate the consumption of specific sugar subtypes and their association with other relevant diseases including various types of cancer. In addition, a possible protective effect of fruit juice should be investigated in further studies.

### Supplementary Information

**Supplementary Material 1.**

## Data Availability

The data that support the findings of this study are available from UK Biobank but restrictions apply to the availability of these data, which were used under license for Application 53438, and, therefore, are not publicly available. Bona fide researchers can apply to use the UK Biobank dataset by registering and applying at https://www.ukbiobank.ac.uk/enable-your-research/register .

## Abbreviations

BMI: Body mass index
CVD: Cardiovascular disease
FS: Free sugars
g/d: Grams per day
HR: Hazard ratio
ICD-10: International classification of disease 10^th^ revision
IHD: Ischemic heart disease
kJ: Kilojoules
%E: Percentage total energy
SD: Standard deviation
WHO: World Health Organization
WHR: Waist-to-hip-ratio
