# Supplementary material for: Association of sugar intake from different sources with cardiovascular disease incidence in the prospective cohort of UK Biobank participants
Source: Nutr J. 2024 Feb 22;23:22. doi: 10.1186/s12937-024-00926-4 (PMC10882929; doi:10.1186/s12937-024-00926-4)
Supplement: Supplementary file 1 — Supplementary Material 1. [file 12937_2024_926_MOESM1_ESM.pdf]

Supplementary Material

Nutrition Journal

Association of sugar intake from different sources with cardiovascular disease incidence in the prospective cohort of UK Biobank participants

Sylva M Schaefer, M.Sc.[1#]; Anna Kaiser, M.Sc.[1#*]; Gerrit Eichner, PhD[2§]; Mathias Fasshauer, MD[1,3§]

1. Institute of Nutritional Science, Justus-Liebig University of Giessen, Giessen, Germany.
2. Mathematical Institute, Justus-Liebig University of Giessen, Giessen, Germany.
3. Center for Sustainable Food Systems, Justus-Liebig University of Giessen, Giessen, Germany.

[#]SMS and AK contributed equally to this work and are joint first authors.
[§]GE and MF contributed equally to this work and are joint senior authors.
*Corresponding author. Mailing address: Goethestr. 55, 35390 Giessen, Germany. Phone: +49 641 9939067. E-mail: sylva.schaefer@ernaehrung.uni-giessen.de

Index

Figure S1

Sugar sources relevant to the present study

Abbreviations: FS, Free sugars

Figure S2

Flowchart of participant selection

The following exclusion criteria were applied to all analyses: 1) missing lifestyle risk factors (physical activity or smoking status), 2) diagnosis of ischemic heart disease (IHD) or stroke before completion of the last Oxford WebQ (=pre-existing CVD, Cardiovascular disease), 3) missing socio-economic factors (Townsend deprivation index, total household income, ethnic background, highest qualification, or overall health rating), 4) missing data of the physical exam (body mass index (BMI), systolic blood pressure (SBP)), 5) pre-existing malabsorption or diabetes, 6) current pregnancy or the possibility of being pregnant, and 7) implausible energy or carbohydrate intake, i.e., 0 kJ/d intake on at least one occasion, being in the upper 0.1 % of total energy and/or carbohydrate consumption or total energy intake <1.1 × basal metabolic rate - 500 kcal (under-reporting) or >2.5 × basal metabolic rate + 500 kcal (over-reporting). Basal metabolic rate was defined according to the Oxford equation (1).

Figure S3

Directed acyclic graph

Directed acyclic graph representing the assumed relationships between variables that may affect the relationship between sugar intake (green disc with inscribed triangle; exposure) and risk of CVD (blue disc with inscribed vertical bar; outcome). Other blue discs denote ancestors of the outcome, i.e., direct or indirect predecessors of the outcome; white discs denote covariates for which the model was adjusted, i.e., age, energy intake, highest qualification, physical activity (MET per week), sex, and smoking. Grey discs denote other variables and pink discs denote ancestors of both exposure and outcome. Green arrows indicate causal paths.

Abbreviations: BMI, Body mass index; CVD, Cardiovascular disease; MET, Metabolic equivalent of task

Figure S4

Landmark analysis

Association of (a) FS, (b) intrinsic sugars, as well as FS in (c) beverages, (d) solids, (e) soda/fruit drinks, (f) juice, (g) milk-based drinks, (h) tea/coffee, (i) treats, (j) cereals, (k)

toppings, and (l) sauces (all %E) with CVD risk (landmark analysis; n=175,789; number of events=11,991). Models are adjusted for age, energy intake, highest qualification, physical activity, sex, and smoking status. Covariates not fulfilling the proportional hazard assumption are stratified. The vertical line indicates the nadir. Abbreviations: CVD, Cardiovascular disease; FS, Free sugars; HR, Hazard ratio

Figure S5

Unintentional weight loss removed

Association of (a) FS, (b) intrinsic sugars, as well as FS in (c) beverages, (d) solids, (e) soda/fruit drinks, (f) juice, (g) milk-based drinks, (h) tea/coffee, (i) treats, (j) cereals, (k) toppings, and (l) sauces (all %E) with CVD risk (unintentional weight loss removed; n=148,640; number of events=10,315). Models are adjusted and presented as indicated in Figure S4. Abbreviations: CVD, Cardiovascular disease; FS, Free sugars; HR, Hazard ratio

Figure S6

Non-typical diet removed

Association of (a) FS, (b) intrinsic sugars, as well as FS in (c) beverages, (d) solids, (e) soda/fruit drinks, (f) juice, (g) milk-based drinks, (h) tea/coffee, (i) treats, (j) cereals, (k) toppings, and (l) sauces (all %E) with CVD risk (non-typical diet removed; n=117,966; number of events=8,732). Models are adjusted and presented as indicated in Figure S4. Abbreviations: CVD, Cardiovascular disease; FS, Free sugars; HR, Hazard ratio

Figure S7

First Oxford WebQ only

Association of (a) FS, (b) intrinsic sugars, as well as FS in (c) beverages, (d) solids, (e) soda/fruit drinks, (f) juice, (g) milk-based drinks, (h) tea/coffee, (i) treats, (j) cereals, (k) toppings, and (l) sauces (all %E) with CVD risk. Only the first Oxford WebQ was used for intake estimation (n=176,352; number of events=12,355). Models are adjusted and presented as indicated in Figure S4. Abbreviations: CVD, Cardiovascular disease; FS, Free sugars; HR, Hazard ratio

Figure S8

Adjustment for diet quality score

Association of (a) FS, (b) intrinsic sugars, as well as FS in (c) beverages, (d) solids, (e) soda/fruit drinks, (f) juice, (g) milk-based drinks, (h) tea/coffee, (i) treats, (j) cereals, (k) toppings, and (l) sauces (all %E) with CVD risk. Models were further adjusted for diet quality score (n=174,164; number of events=12,145). Models are adjusted and presented as

indicated in Figure S4. Abbreviations: CVD, Cardiovascular disease; FS, Free sugars; HR, Hazard ratio

Figure S9

Only participants with >1 Oxford WebQ

Association of (a) FS, (b) intrinsic sugars, as well as FS in (c) beverages, (d) solids, (e) soda/fruit drinks, (f) juice, (g) milk-based drinks, (h) tea/coffee, (i) treats, (j) cereals, (k) toppings, and (l) sauces (all %E) with CVD risk. Only participants who completed more than one Oxford WebQ were included in the analysis (n=109,316; number of events=7,124). Models are adjusted and presented as indicated in Figure S4. Abbreviations: CVD, Cardiovascular disease; FS, Free sugars; HR, Hazard ratio

Figure S10

IHD + Stroke

Association of FS (a + b), intrinsic sugars (c + d), FS in beverages (e + f), FS in solids (g + h), FS in beverage subtypes (i to p) and FS in solids subtypes (q to x) (all %E) with IHD (n=176,929; number of events=9,950) and stroke (n=179,082; number of events=3,066). Models are adjusted and presented as indicated in Figure S4. Covariates not fulfilling the proportional hazard assumption are stratified. Abbreviations: FS, Free sugars; HR, Hazard ratio; IHD, Ischemic heart disease

Table S1

Overview of main results

Linear (p^{lin}) and non-linear ($p^{non\text{-}lin}$) p-values for associations with CVD, the nadir, as well as HRs (95% confidence intervals) at 0 %E of respective sugar (HR^0), and shape of the curve in case of significance. Abbreviations: CVD, Cardiovascular disease; %E; Percent total energy; FS, Free sugars; HR, Hazard ratio; NA, not applicable since non-significant results for p^{lin} and $p^{non\text{-}lin}$

References

1. Henry CJK. Basal metabolic rate studies in humans: measurement and development of new equations. Public Health Nutr 2005; 8(7A):1133–52.

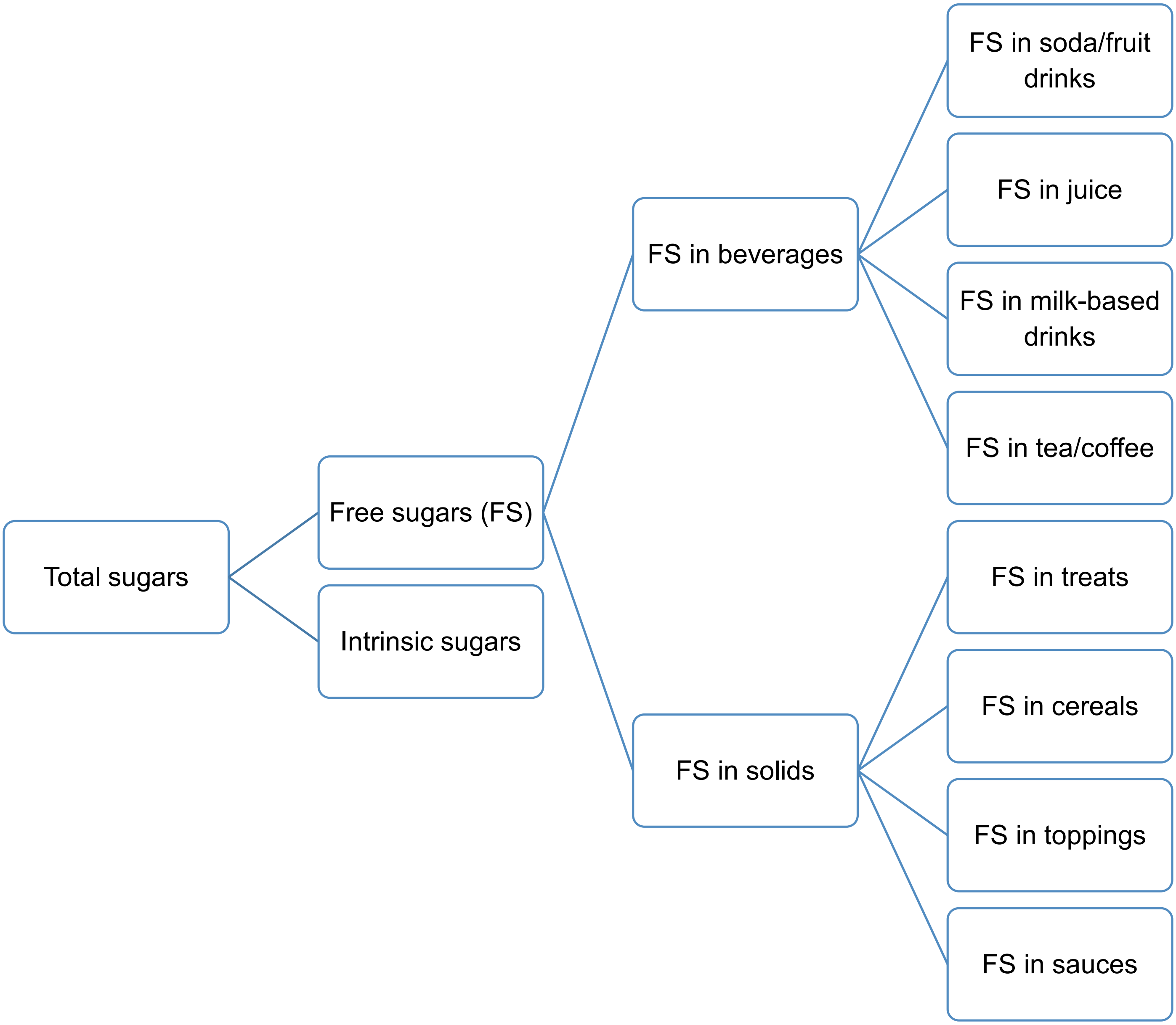

Figure S1 Sugar sources relevant to the present study

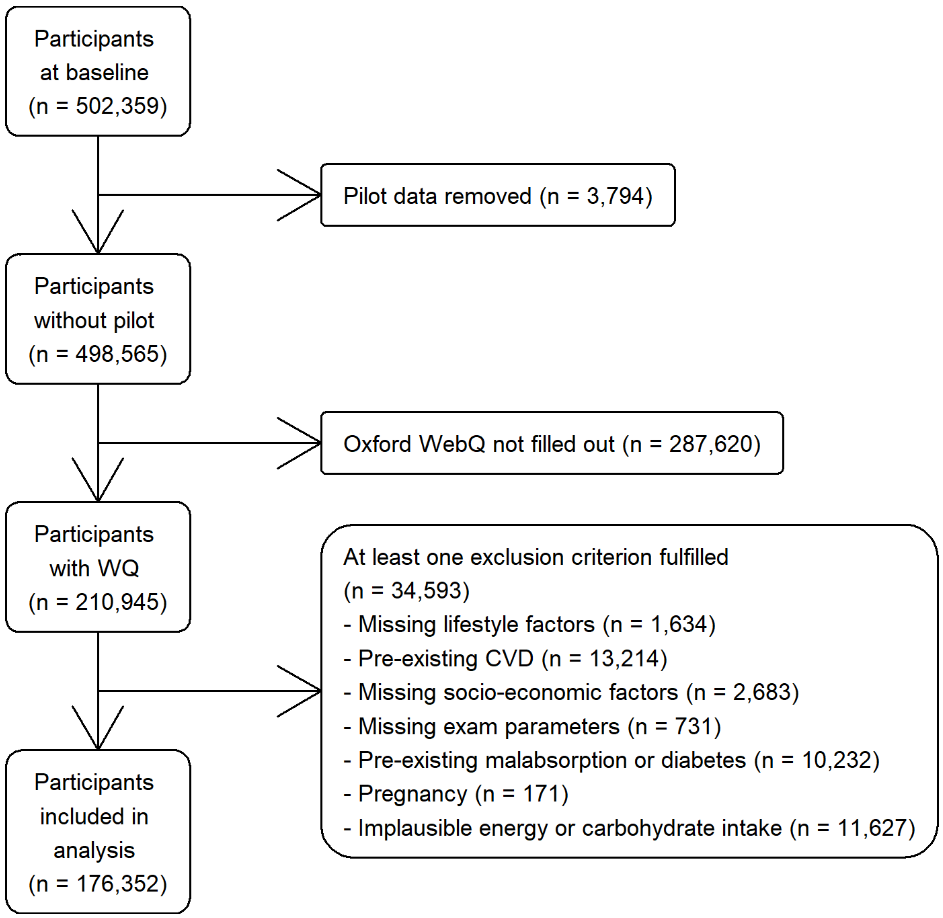

Figure S2 Flowchart of participant selection

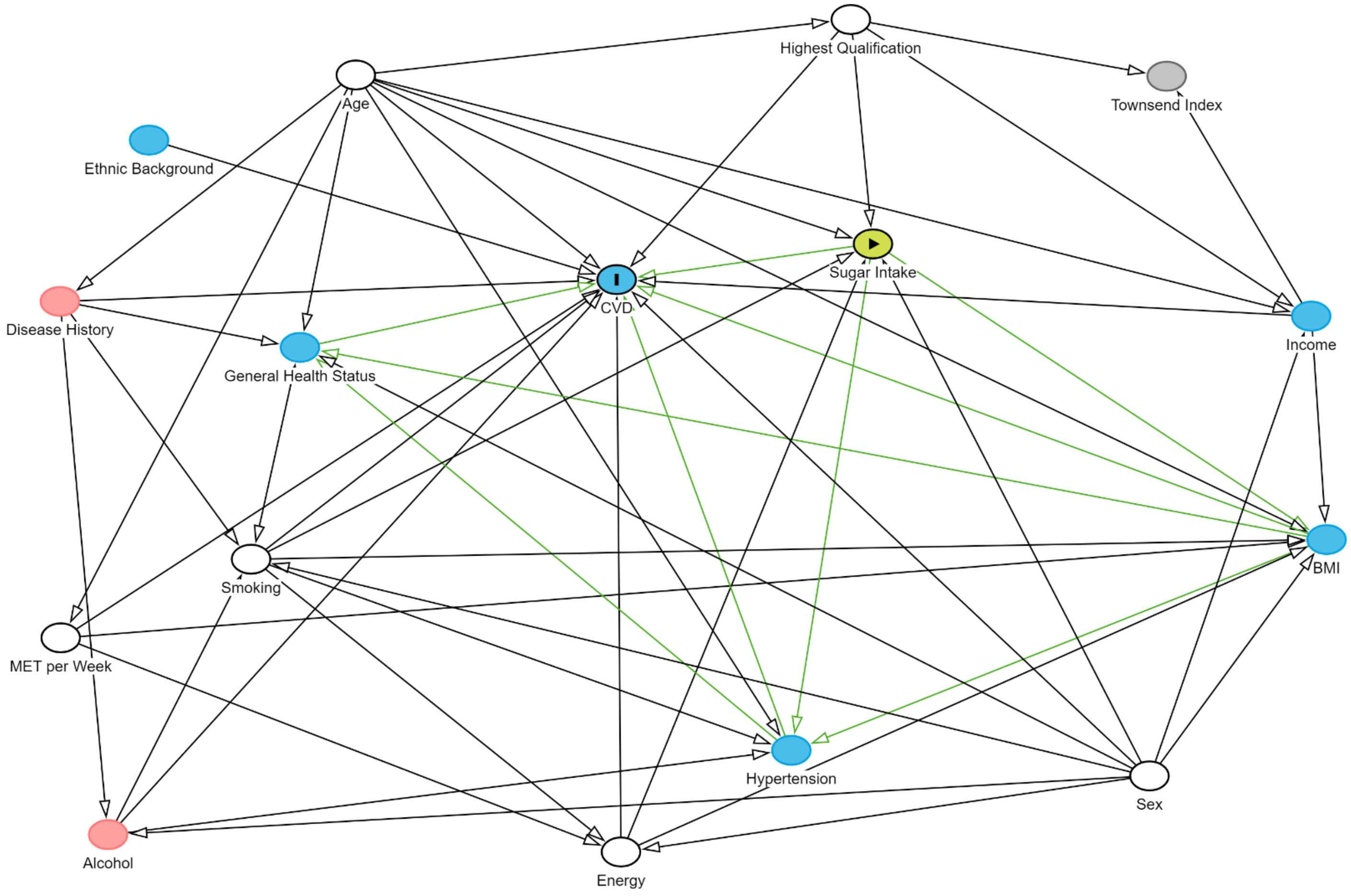

Figure S3 Directed acyclic graph

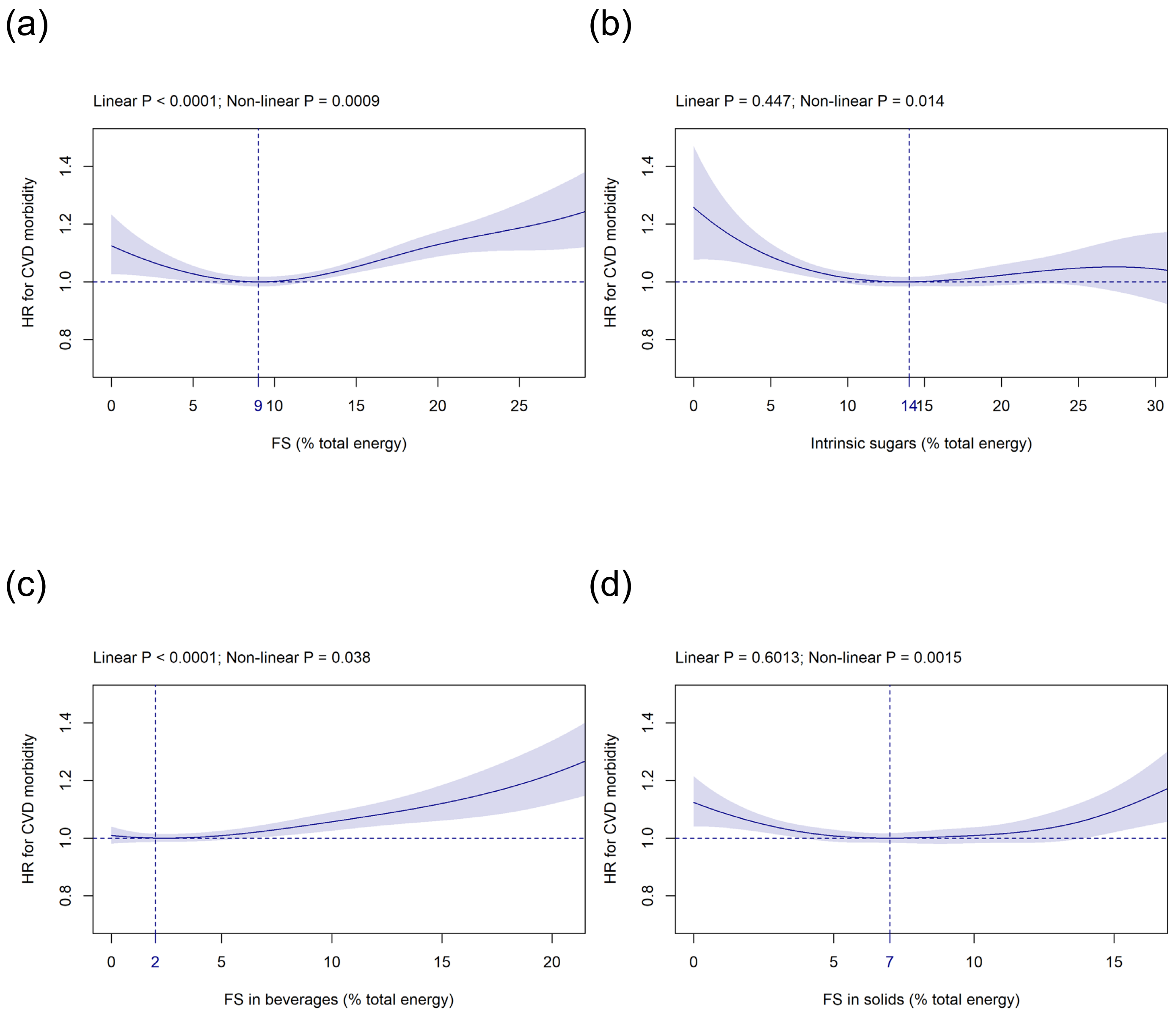

Figure S4 Landmark analysis

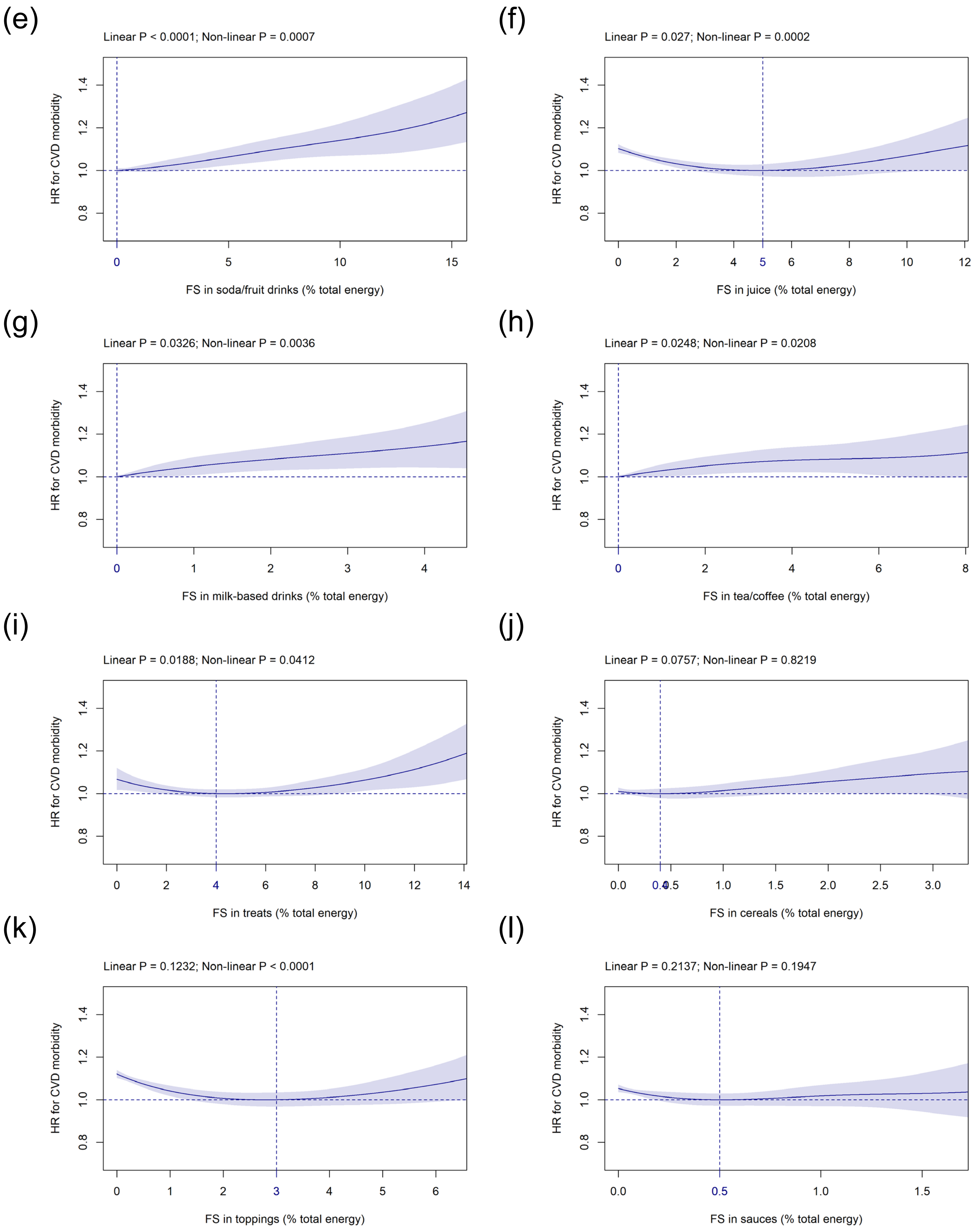

Figure S4 Landmark analysis continued

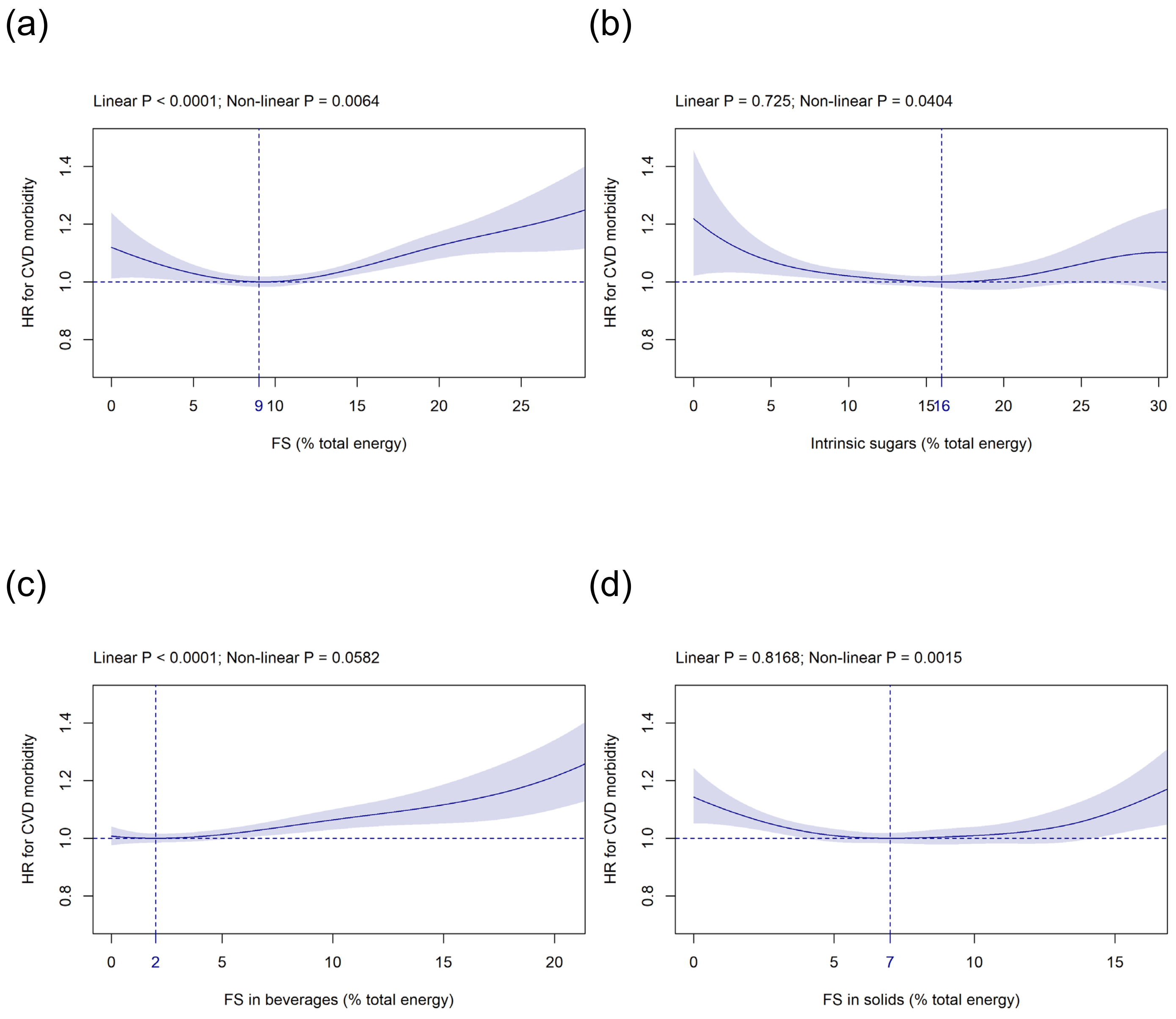

Figure S5 Unintentional weight loss removed

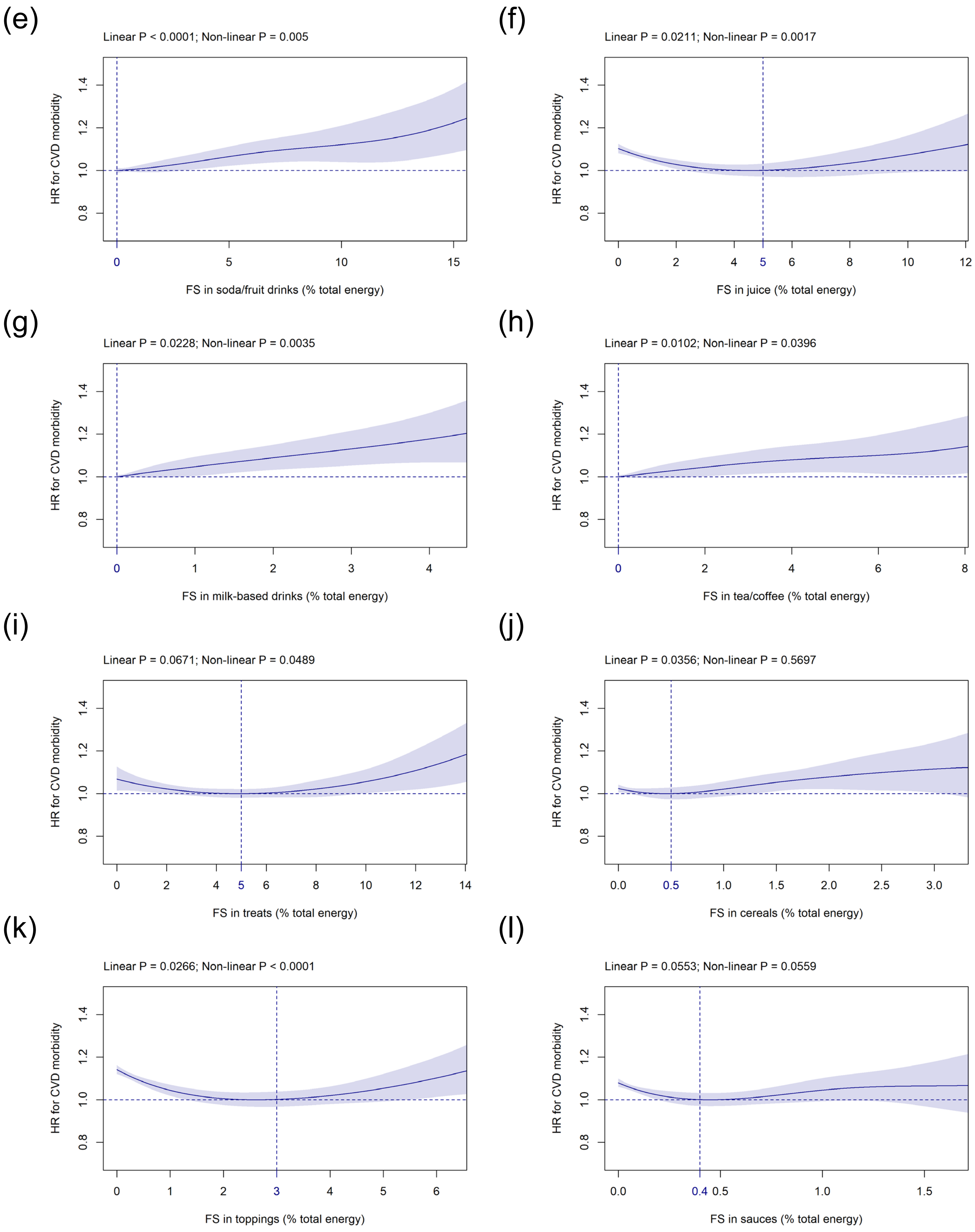

Figure S5 Unintentional weight loss removed continued

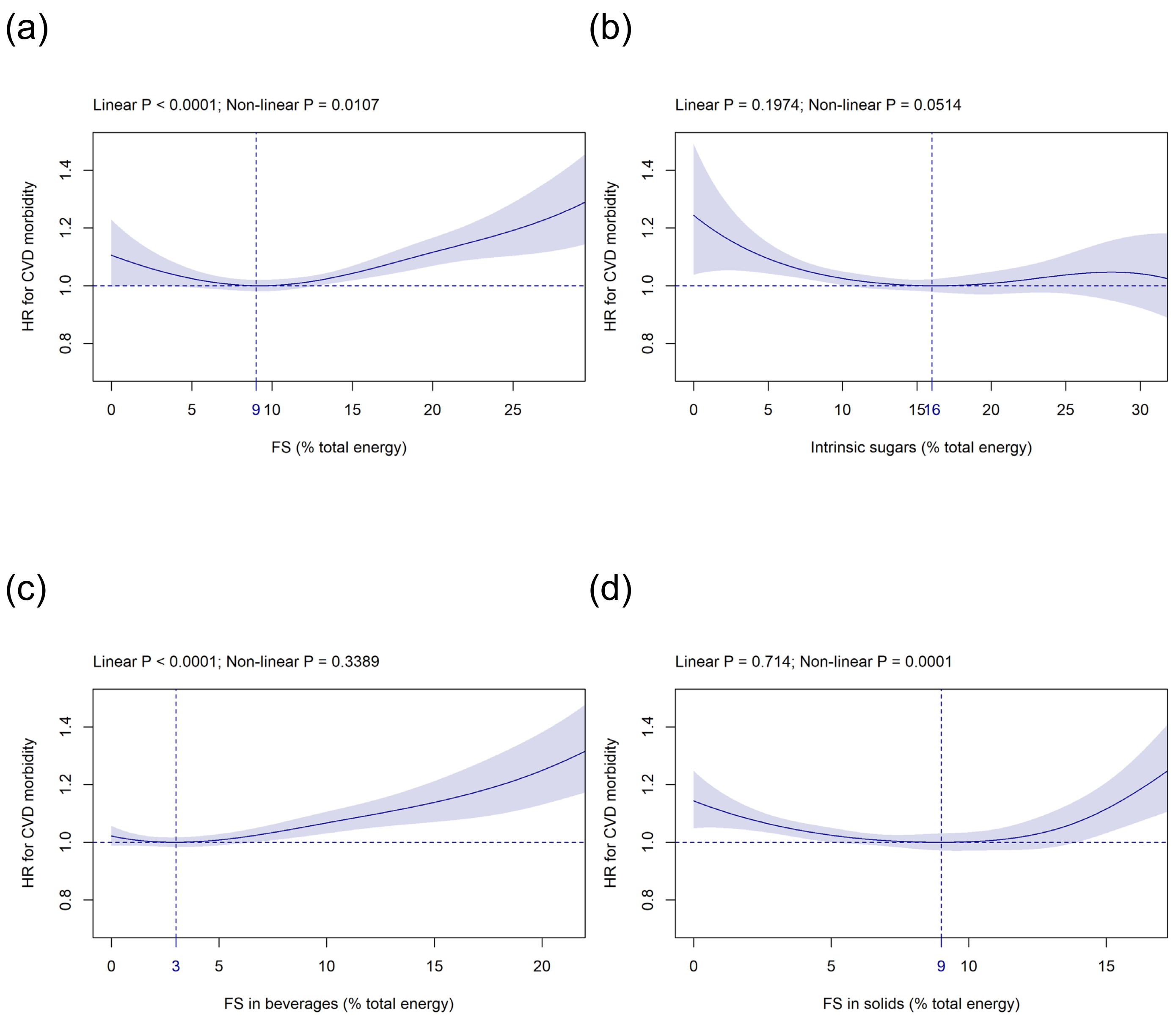

Figure S6 Non-typical diet removed

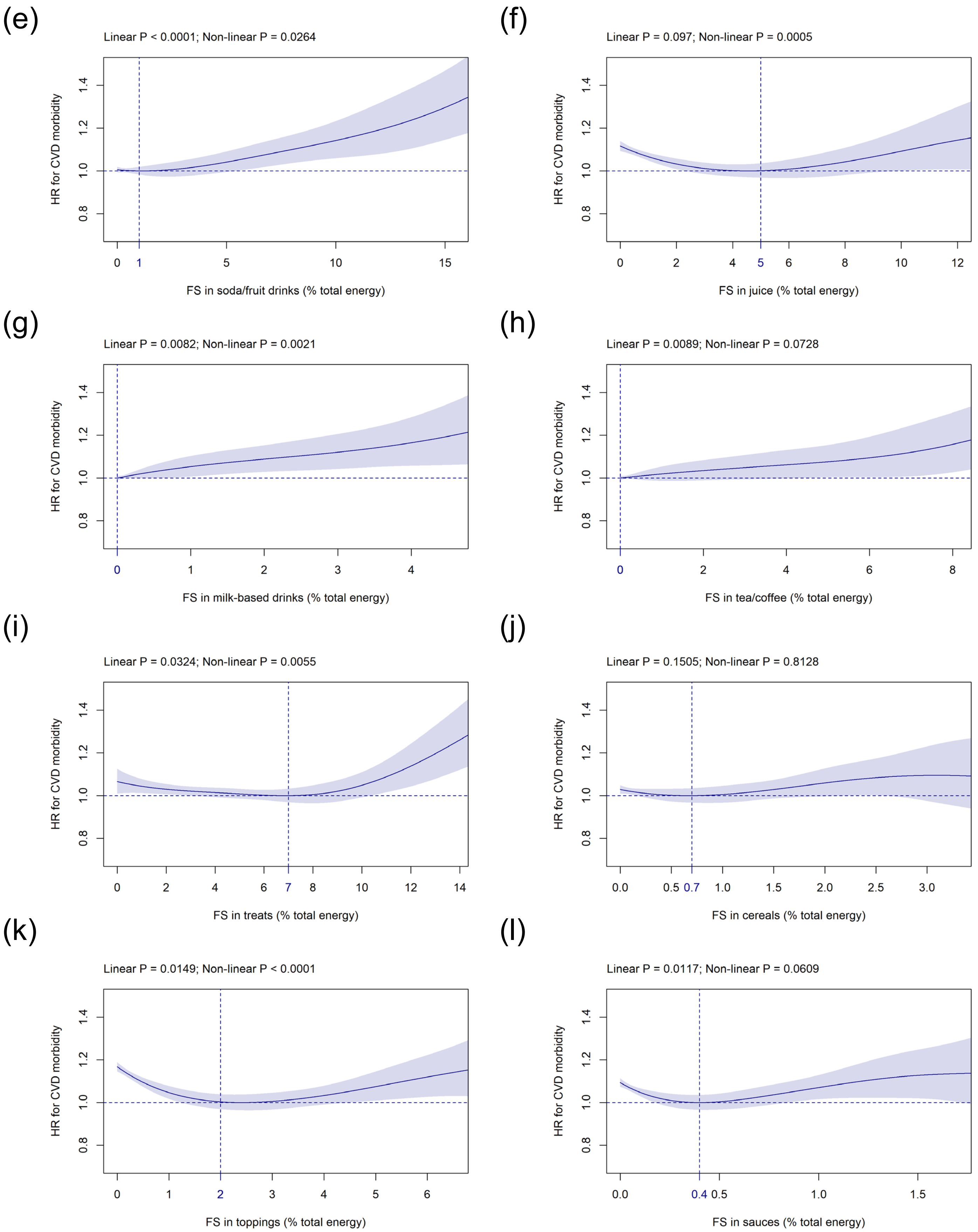

Figure S6 Non-typical diet removed continued

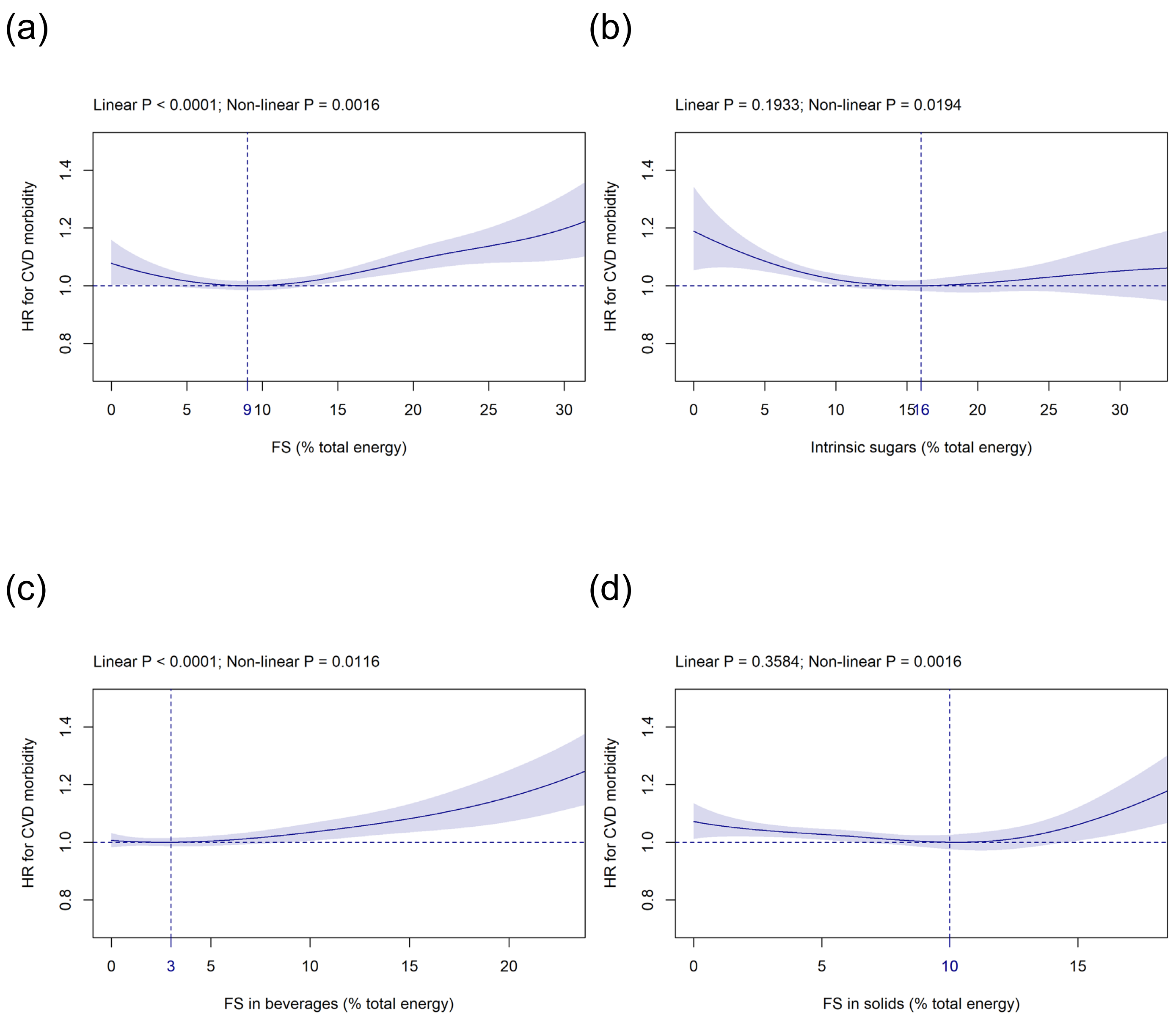

Figure S7 First Oxford WebQ only

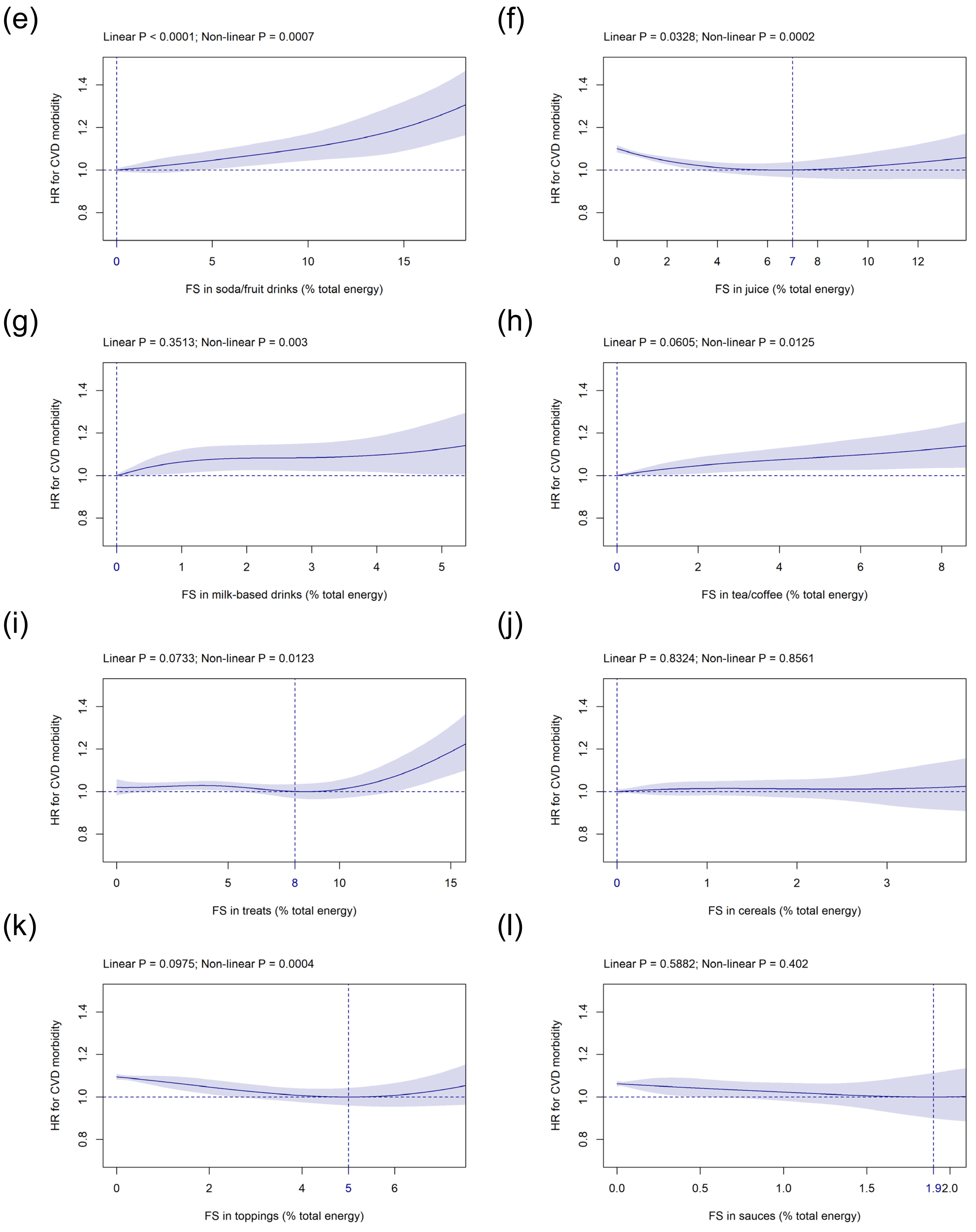

Figure S7 First Oxford WebQ only continued

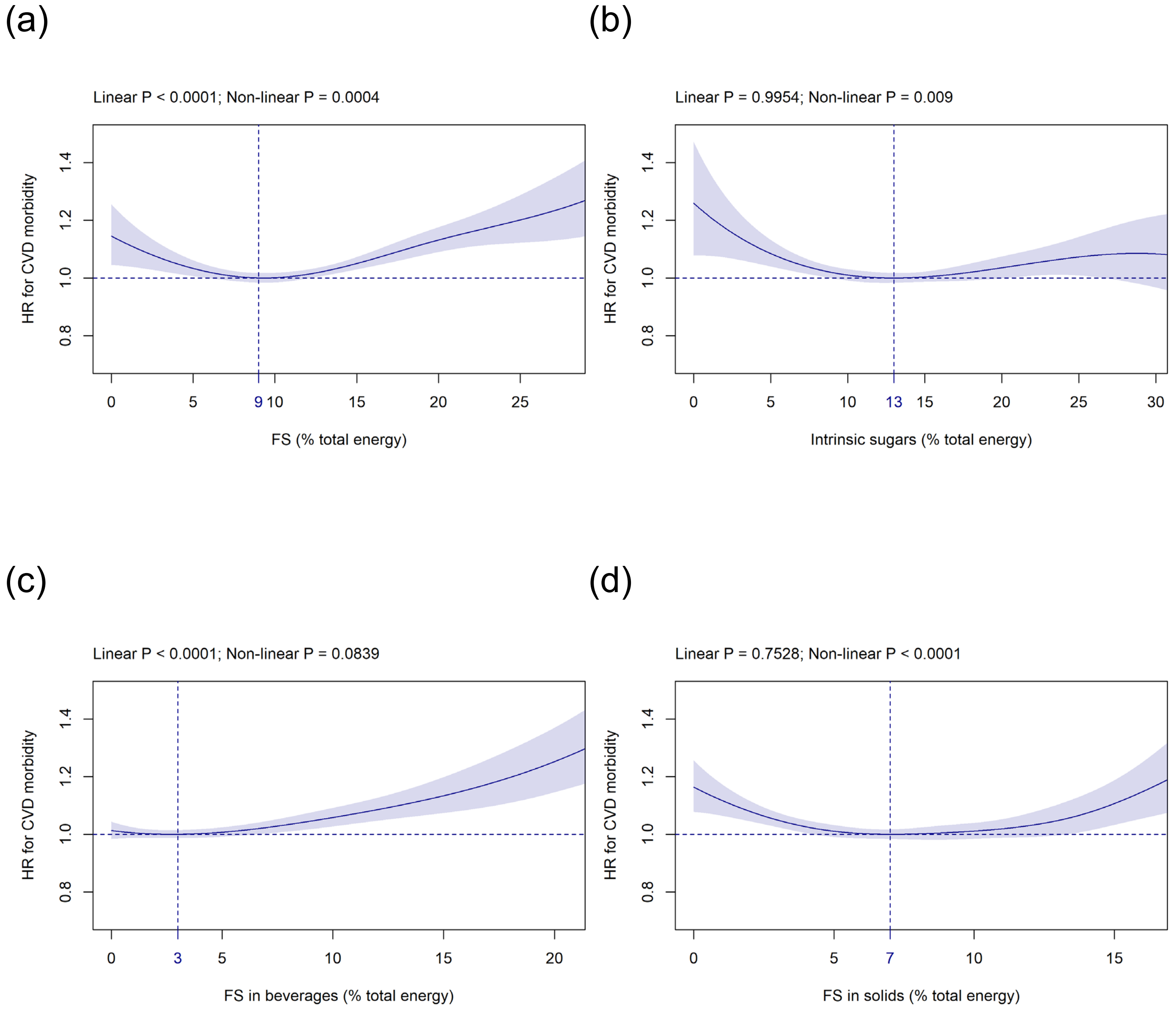

Figure S8 Adjustment for diet quality score

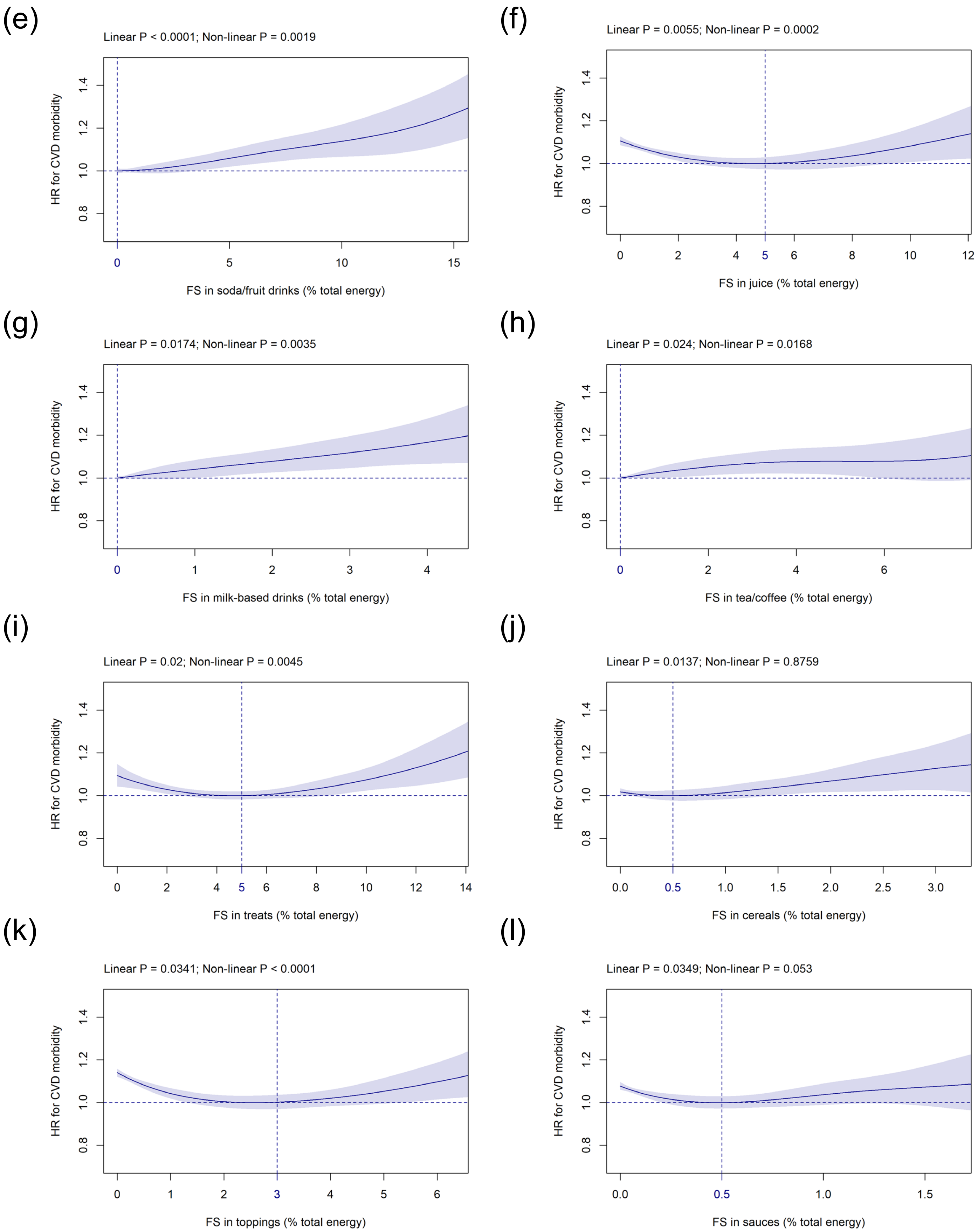

Figure S8 Adjustment for diet quality score continued

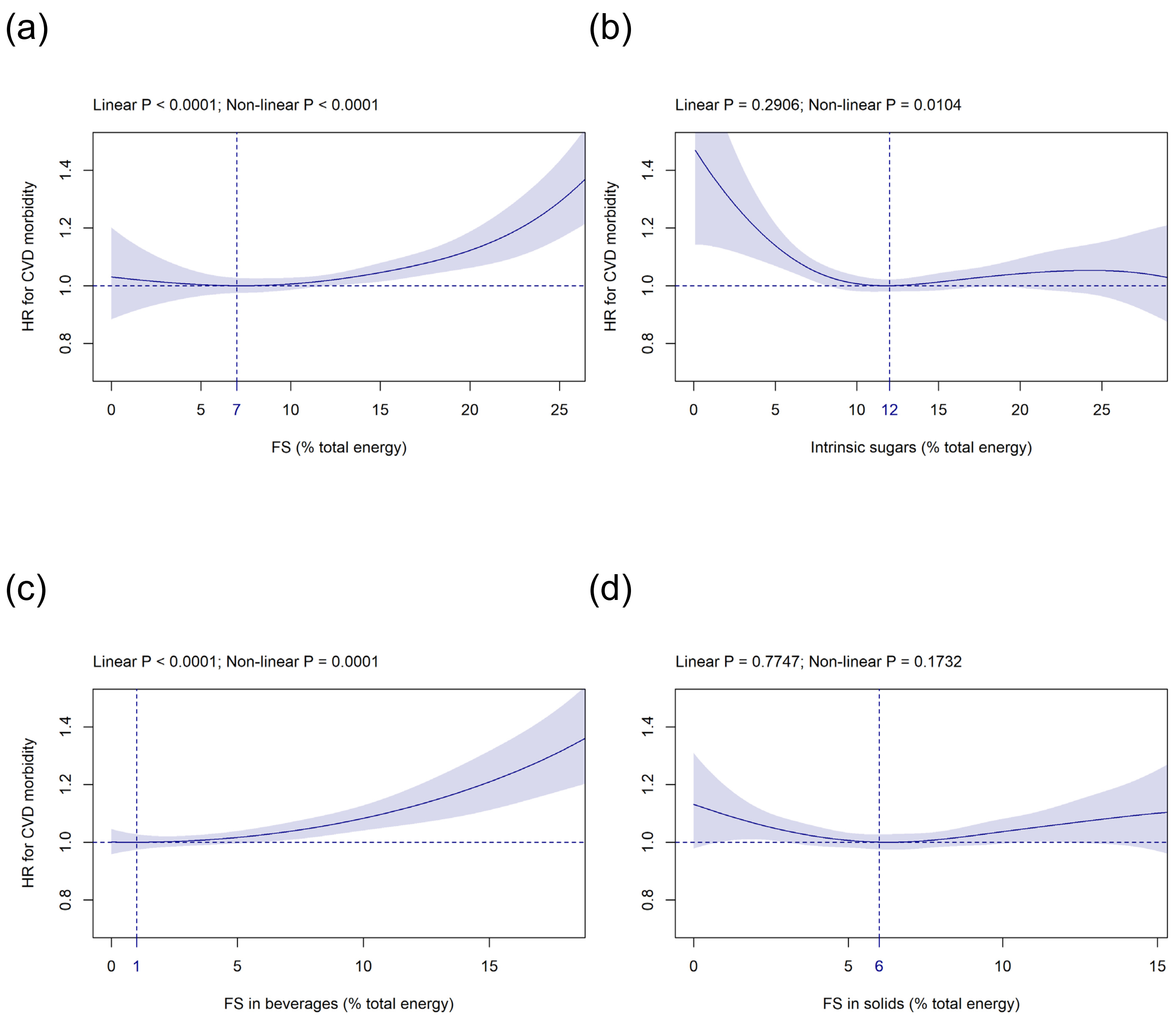

Figure S9 Only participants with >1 Oxford WebQ

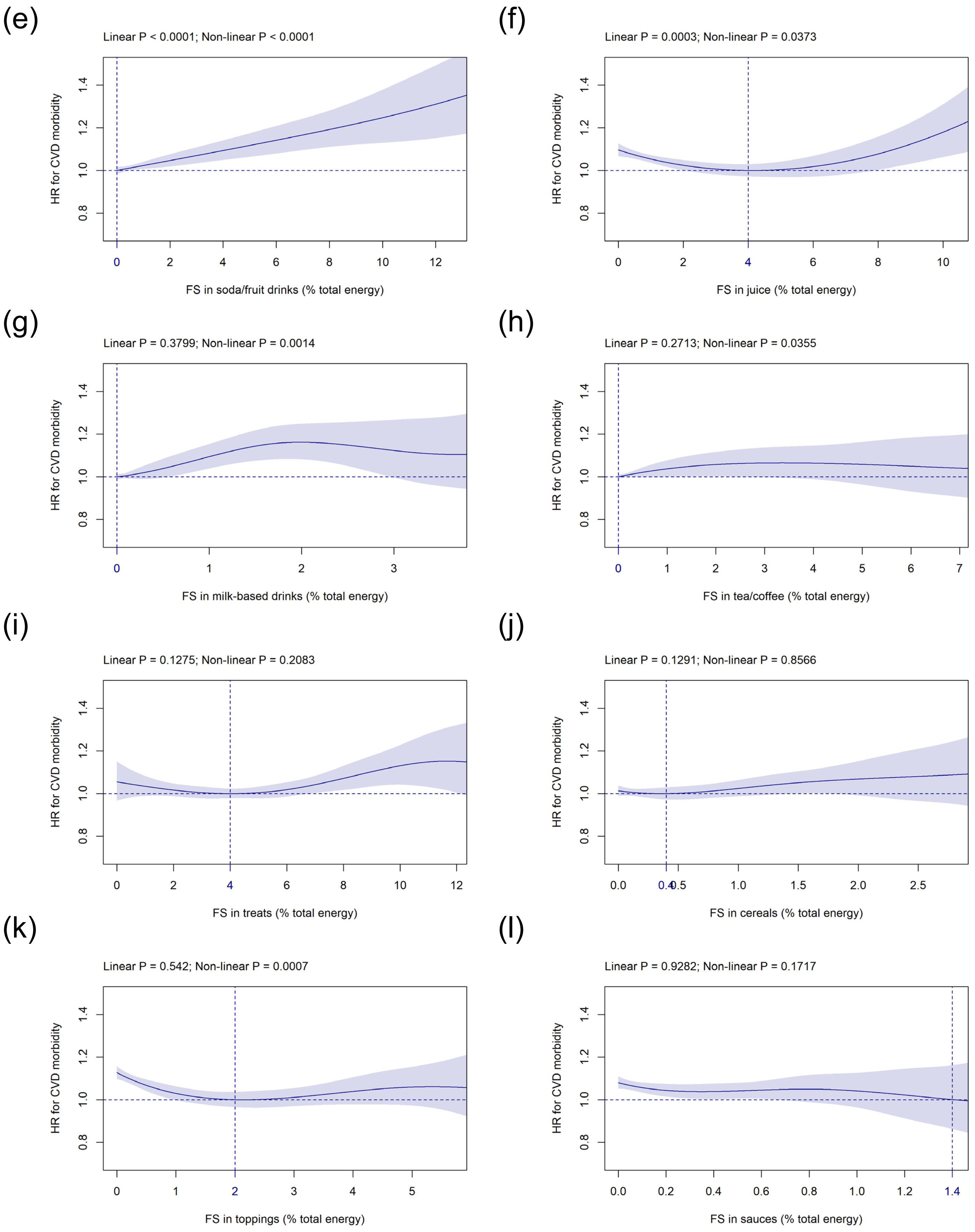

Figure S9 Only participants with >1 Oxford WebQ continued

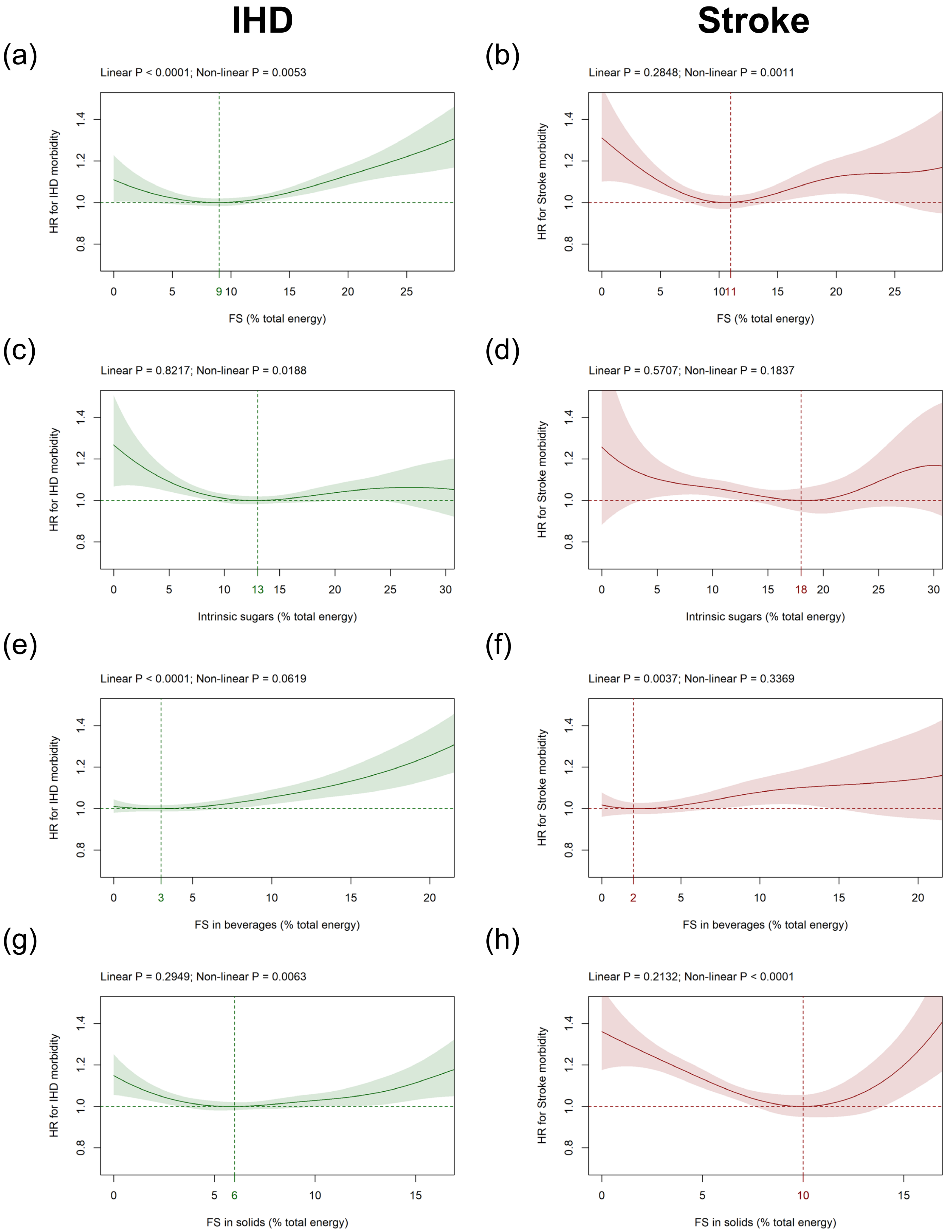

Figure S10 IHD + Stroke

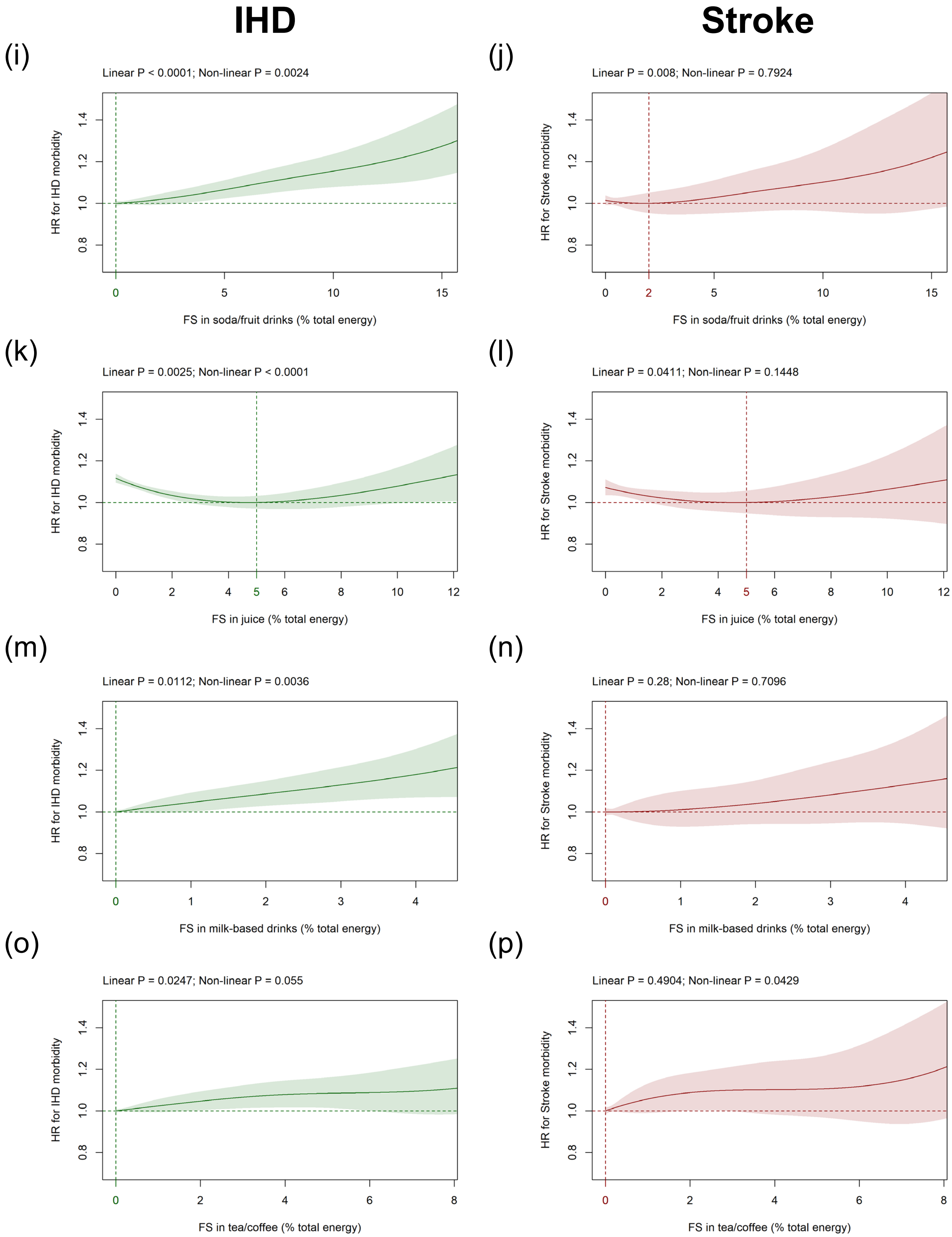

Figure S10 IHD + Stroke continued

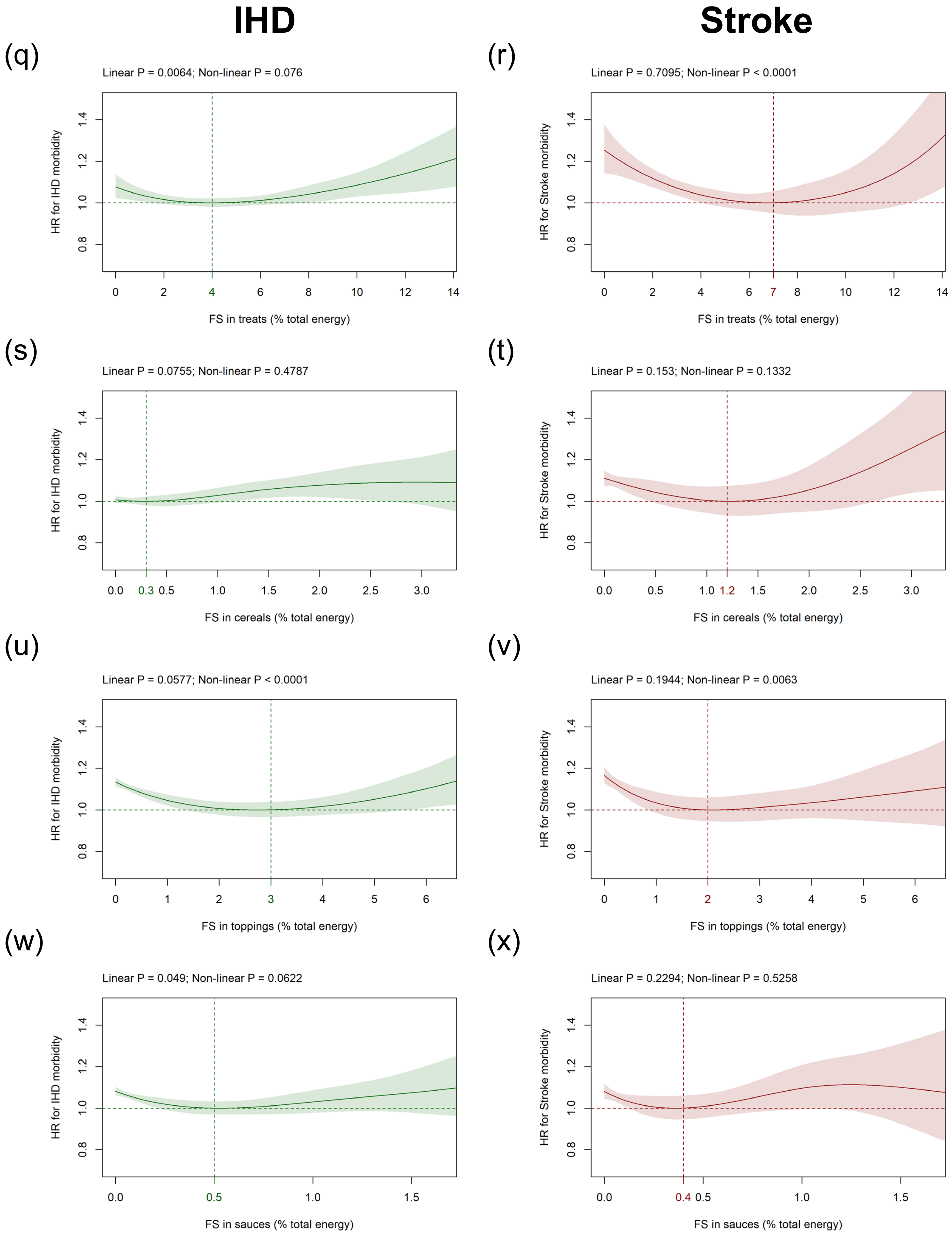

Figure S10 IHD + Stroke continued

Table S1 Overview of main results[1]

**Type of sugar (%E)**	**p^{lin} / $p^{non-lin}$**	**Nadir (%E)**	**HR^0**	**Shape**
FS	**<0.0001** / **0.0001**	9	1.15 (1.05, 1.26)	J-shaped
Intrinsic sugar	0.5032 / **0.0104**	14	1.26 (1.08 1.47)	Non-linear descending
FS in beverages	**<0.0001** / 0.0669	3	1.01 (0.99, 1.04)	Linear
FS in solids	0.6186 / **<0.0001**	7	1.16 (1.07, 1.25)	U-shaped
FS in soda/fruit drinks	**<0.0001** / 0.**0028**	0	1.00 (0.99, 1.01)	Linear
FS in juice	0.0117 / **<0.0001**	5	1.11 (1.09, 1.13)	U-shaped
FS in milk-based drinks	**0.0088** / **0.0049**	0	1.00 (0.99, 1.01)	Linear
FS in tea/coffee	0.**0162** / **0.0116**	0	1.00 (0.99, 1.01)	Linear
FS in treats	0.**0107** / 0.**0049**	5	1.09 (1.04, 1.14)	J-shaped
FS in cereals	**0.0148** / 0.8334	0.5	1.02 (1.00, 1.03)	Linear
FS in toppings	**0.0342** / **<0.0001**	3	1.14 (1.12, 1.16)	U-shaped
FS in sauces	**0.0358** / **0.0346**	0.5	1.08 (1.06, 1.10)	U-shaped

[1]Linear (p^{lin}) and non-linear ($p^{non-lin}$) p-values for associations with CVD, the nadir, as well as HRs (95% confidence intervals) at 0 %E of respective sugar (HR^0), and shape of the curve in case of significance. Abbreviations: CVD, Cardiovascular disease; %E; Percent total energy; FS, Free sugars; HR, Hazard ratio
